# Screening and Stability Analysis of Reference Genes for Gene Expression Normalization in Hybrid Yellow Catfish (*Pelteobagrus fulvidraco* ♀ × *Pelteobagrus vachelli* ♂) Fed Diets Containing Different Soybean Meal Levels

**DOI:** 10.1155/2023/1232518

**Published:** 2023-09-22

**Authors:** Chaohui Guo, Zihao Zhang, Meina Zhang, Guojun Guo, Guangqing Yu, Daoquan Zhao, Ming Li, Guoxi Li, Bianzhi Liu

**Affiliations:** ^1^College of Animal Science and Technology, Henan Agricultural University, Zhengzhou 450046, China; ^2^Henan University of Animal Husbandry and Economy, Zhengzhou 450046, China; ^3^Henan Academy of Fishery Sciences, Zhengzhou 450044, China; ^4^Yiluo River Field Scientifical Observation and Reseaarch Station of Aquatic Animals in Yellow River Basin, Lushi 472200, China

## Abstract

In this study, we screened the expression stability of six reference genes (*18S rRNA*, *β-actin*, *GAPDH*, *EF1a*, *B2M*, and *HPRT1*) in hybrid yellow catfish (*n* = 6), considering the SBM levels, sampling time points, and different tissues. Four different statistical programs, BestKeeper, NormFinder, Genorm, and Delta Ct, combined with a method that comprehensively considered all results, were used to evaluate the expression stability of these reference genes systematically. The results showed that SBM levels significantly impacted the expression stability of most of the reference genes studied and that this impact was time-, dose-, and tissue-dependent. The expression stability of these six reference genes varied depending on tissue, sampling time point, and SBM dosage. Additionally, more variations were found among different tissues than among different SBM levels or sampling time points. Due to its high expression, *18S rRNA* was excluded from the list of candidate reference genes. *β-actin* and *GAPDH* in the liver and *β-actin*, *HPRT1* and *EF1a* in the intestine were the most stable reference genes when SBM levels were considered. *HPRT1*, and *EF1a* in tissues sampled at 2 W and EF1a and *β*-actin in tissues sampled at 4 and 6 W were proposed as two stable reference genes when different tissues were considered. When the sampling time points were considered, *β*-actin, EF1a, and HPRT1 were the top three stable reference genes in the intestine. In contrast, *β*-actin and B2M are the most stable reference genes in the liver. In summary, *β*-actin, EF1a, and HPRT1 were the more stable reference genes in this study. The stability of reference genes depends on the tissues, sampling time points, and SBM diet levels in hybrid yellow catfish. Therefore, attention should be paid to these factors before selecting suitable reference genes for normalizing the target genes.

## 1. Introduction

Gene expression level is often considered an essential indicator of an organism's biological functions in all branches of biological studies. Hence, the precise monitoring of gene expression is vital for analyzing target gene expression patterns. Quantitative real-time PCR (RT-qPCR), known for its convenience, sensitivity, and specificity, is the most widely used tool for analyzing the expression of target genes in various studies, including molecular nutriology. In RT-qPCR, the expression of the target gene should be standardized and normalized against a reference gene to reduce experimental error and ensure the accuracy of the experiment [1, 2]. Therefore, appropriate reference genes with stable expression across all experimental samples were required. Unfortunately, there is growing evidence that, with an invariable expression theory, assumed reference genes traditionally involved in structural functions or primary cell metabolism frequently vary with experimental conditions, such as developmental stage, physical conditions, and nutritional status [3, 4]. Consequently, errors could result if inappropriate reference genes were used as normalizers [5]. The stability of reference gene expression must be assessed before its use in RT-qPCR analysis.

With the urgent need for more sustainable aquaculture production and the sharp rise in fish meal (FM) prices caused by dwindling marine fish stocks, fish feed containing alternative protein sources to FM is in high demand [6–8]. Consequently, plant protein, the most cost-effective and widely used substitute represented by soybean meal (SBM), has been widely used in fish feed [9–11]. Meanwhile, adverse effects, most associated with enteritis (i.e., SBM-induced enteritis, SBMIE), progress to a pathological situation characterized by shortened mucosal folds and swollen lamina propria and subepithelial mucosa, or intense infiltration of various inflammatory cells, are often occurring in a species-, time-, or dose-dependent manner [9, 12, 13]. Ruined gastrointestinal barriers result in destructive consequences for fish homeostasis [13]. Thus, negative effects on growth performance [14], nutrient-sensing and metabolic responses [15], development, and reproduction [16] were all involved.

Exploring the potential molecular mechanisms underlying this lesion is critical for developing effective strategies to treat enteritis while improving fish immunity and plant protein utilization. However, it is essential to note that the degree of hazard caused by plant proteins depends on both the dosage and duration of exposure [10], and significant differences in plant protein sensitivity are commonly observed among the different feeding habits of fish species [17, 18]. It is generally believed that the progressive characteristic consists of the course of the exposure, including histopathological changes and expression patterns of genes involved in immunoreaction or nutrient intake, due to the dynamic regulation of nutritional and immunoregulatory physiology. For example, enteritis caused by SBM in grass carp (*Ctenopharyngodon idellus*), the herbivorous species, is milder and has more active and faster immunomodulation, a shorter inflammatory process, and faster recovery than omnivorous or carnivorous species, and the expression patterns of immune-associated genes vary during incubation, prodromal, symptomatic, or convalescent periods [10].

To our knowledge, only a few investigations have focused on assessing the expression stability of reference genes in fish fed a plant-based diet, including SBM. However, numerous studies have shown that the expression stability of reference genes varies between treatments [19–22]. Furthermore, misinterpretation of the data caused by incorrect normalization of the reference genes frequently occurs. Hence, systematic evaluation and screening of the stability of reference genes in fish-fed plant-based diets are necessary to investigate the biological mechanisms of plant-based diet intolerance.

Hybrid yellow catfish, one of the most important breeding species in China [23], can be used as an ideal model test organism for SBMIE because of its unusual dose- and time-dependent enteritis caused by SBM [17, 24]. Therefore, this study investigated the expression stability of the six candidate reference genes in hybrid yellow catfish-fed diets with varying SBM levels. The tissue-, time-, and dose-dependent characteristics and expression stability of these reference gene expressions were assessed and evaluated in four tissues using RT-qPCR. Expression stability was verified by normalizing the expression of *MLKL* (mixed lineage kinase domain-like *MLKL*). Our findings provide a foundation for accurately normalizing target gene expression to further investigate the biological mechanisms of SBM-induced nutritional and metabolic diseases.

## 2. Materials and Methods

### 2.1. Fish Management and Experimental Procedures

Five isonitrogenous and isolipid experimental diets were formulated. A diet containing 40% FM was used as the control group and was designated as 0%. The other four diets, in which 25%, 50%, 75%, and 100% FM were replaced with SBM, were named 25%, 50%, 75%, and 100%, respectively. Hybrid yellow catfish were obtained from a commercial farm in Zhengzhou, Henan Province, China, and transported to the outdoor aquaculture system of the Henan Fisheries Research Institute. The experimental diets are listed in Supplemental Table S1.

After 1 week of acclimation, 750 fish (initial body weight: 9.7 ± 0.5 g) were selected and randomly distributed into 15 net cages (size: 1 × 1 × 1.3 m, 1,300 L) installed in the cement pond after 24 hr starvation, with three net cages per treatment. During the experiment, the water temperature ranged from 25 to 28°C; dissolved oxygen was kept above 6.5 mg/L, pH was between 7.5 and 8.5; total ammonia nitrogen was below 0.2 mg/L; and the photoperiod was maintained under natural light. The fish were fed to apparent satiation twice daily (07 : 00 and 18 : 30), and the experiment lasted for 42 days.

After the start of the experiment, samples were collected at weeks 2, 4, and 6. After 24 hr of starvation, two fish were randomly collected and anesthetized with MS-222 (50 mg/L) in each cage (six biological samples per teatment, *n* = 6). Tissue samples (liver, foregut, midgut, and hindgut) were collected and quickly frozen in liquid nitrogen before being stored at −80°C until analysis.

### 2.2. RNA Isolation and cDNA Synthesis

Total RNA was isolated using the TRIzol Reagent (TransGen Biotech, Beijing, China). The purity and integrity of total RNA were detected using a spectrophotometer (Thermo, USA) and 1% agarose gel electrophoresis. PrimeScript™ RT reagent Kit with gDNA Eraser (Perfect Real Time) (TaKaRa, RR047A, Japan) was used to synthesize cDNA, which was then stored at −20°C for RT-qPCR.

### 2.3. Selection of Potential Reference Genes and Primer Design

Nucleotide sequences of *18S rRNA*, *β-actin*, *GAPDH*, *EF1a*, *B2M*, and *HPRT1* were obtained from NCBI GenBank to design gene-specific primers using Primer-BLAST provided by NCBI (Table 1). Primers were synthesized by Sangon Biotech Co., Ltd. (Shanghai, China). Primer specificity was confirmed by a single-band and expected product with a unique melting peak at the melting curve stage of RT-qPCR. Amplification efficiency was measured using standard curves generated from assays made with five-fold serial dilutions (5^0^, 5^−1^, 5^−2^, 5^−3^, and 5^−4^) of cDNA from pooled target templates. Mean threshold cycle (Ct) values were plotted against the logarithm of the cDNA dilution factor. Primer efficiency was calculated using the regression slope by the following equation: *E* = 10^(−1/slope)^−1.

### 2.4. RT-qPCR

RT-qPCR was performed using TB Green® Premix Ex Taq™ II (TaKaRa, Japan) on a CFX96™ Real-Time System (Bio-Rad, USA). Each reaction mixture was 10 *µ*L volume and consisted of 5 *µ*L TB Green® Premix Ex Taq™ II, 0.3 *µ*L of each primer, 2 *µ*L cDNA (200 ng/*µ*L), and 2.4 *µ*L ddH_2_O. The amplification protocol was 95°C for 10 s, 60°C for 30 s, and 72°C for 30 s. The melting curve was determined by systematically monitoring fluorescence from 65 to 95°C with temperature increments of 0.5°C increase per 5 s. All samples were evaluated using six biological replicates for each group and three technical replicates for each of the samples; a non-template was used as a control.

### 2.5. Expression Stability of the Reference Genes

Four different statistical programs, BestKeeper, NormFinder, Genorm, and Delta Ct, were used to ascertain the expression stability of the candidate reference genes. The BestKeeper program calculates the Ct standard deviation (SD), and more stable gene expression is represented by lower SD values [25]. NormFinder is an ANOVA-based model that calculates the expression stability value by obtaining inter- and intra-group gene expression variations. The lowest value indicated the most significant stability [26]. According to Genorm, each reference gene's expression stability value (M value) was determined based on the average pairwise variation among all tested reference genes, which can be used as an alternative reference gene when the M value is less than 1.5. A lower M value indicates higher expression stability [27]. In addition, GeNorm compared the pairwise variation (V) of the evaluated reference genes with those of the others. The V value of V_n_/V_n + 1_ between the two sequential normalization factors was obtained [20]. When the V value of V_n_/V_n + 1_ was <0.15, the number of optimal reference genes was “*n*”. When the V value of V_n_/V_n + 1_ was >0.15, the optimal number of reference genes was “*n* + 1” [28]. The Delta Ct method first computes the *Δ*Ct value between each reference gene and other reference genes in each sample, then computes the standard deviation (SD) of all sample *Δ*Ct values, and finally computes the average SD of each reference gene compared to other reference genes. The lowest average SD value indicates the highest stability [29].

Finally, considering that the basic mathematical principles of each program are different, the final ranking was conducted using a method that comprehensively considered all results, as described in the study by Chen et al. [30]. In short, we computed the geometric average value of the ranking of each reference gene using the four analysis programs. The lowest geometric average value indicated the highest stability.

### 2.6. Validation of Reference Genes

Based on the analysis results at different SBM levels (Table 2). *MLKL* (mixed lineage kinase domain-like) gene expression in tissues of hybrid yellow catfish fed with 6 W SBM-based diets was normalized against the two most stable and least stable reference genes (*β-actin*, *GAPDH*, and *18S rRNA* for the liver; *EF1a*, *β-actin*, and *18S rRNA* for foregut; *β-actin*, *B2M*, and *EF1a* for midgut; *β-actin*, *18S rRNA*, *and GAPDH* for hindgut) for validating the candidate reference genes. The 2^−*ΔΔ*Ct^ method was used to determine *MLKL* expression levels.

### 2.7. Data Analysis

Using the logarithm of sample concentration as the independent variable and the Ct value as the dependent variable, calibration curves and the correlation coefficients (*R*^2^) were calculated using Excel 2021. The Shapiro–Wilk test was employed to determine data normality, and Levene's test was used to determine homoscedasticity (*P* < 0.05). The data are expressed as mean ± Standard Deviation (SD) and analyzed using SPSS 26.0, one-way ANOVA, followed by Duncan's multiple comparison test. Statistical significance was set at *P* < 0.05. GraphPad Prism 5 (GraphPad Program, USA) was used to plot the graphs.

## 3. Results

### 3.1. Primer Specificity and PCR Efficiency of Candidate Reference Genes

The amplification efficiency values of the six genes ranged from 95.21% to 114.23%, and *R*^2^ varied from 0.9827 to 0.9973 (Table 1). Melting curve analysis generated only one peak for each amplification (Figure S1). These results demonstrated that these candidate primers were suitable for RT-qPCR analysis.

### 3.2. Expression Levels of Candidate Reference Genes

**Figure 1:**
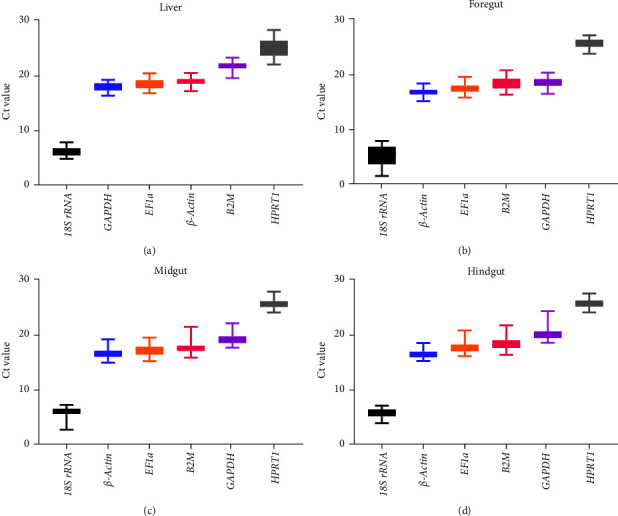
Basic information about the raw Ct values for each candidate reference gene obtained by RT-qPCR in hybrid yellow catfish-fed diets containing different soybean meal levels. The box indicates the 75th and 25th percentiles, and the line across the box depicts the median. Whiskers represent the maximum and minimum values. Each box represents pooled data from all samples of each tissue.

The expression level of each reference gene for all samples, as the mean Ct value, is shown in Figure 1. The six reference genes had Ct values ranging from 4.76 (*18S rRNA*) to 28.15 (*HPRT1*) in the liver, 1.41 (*18S rRNA*) to 26.98 (*HPRT1*) in the foregut, 2.62 (*18S rRNA*) to 27.73 (*HPRT1*) in the midgut, and 3.58 (*18S rRNA*) to 27.43 (*HPRT1*) in the hindgut. Among the four tissues, *18S rRNA* showed the highest expression, whereas *HPRT1* showed the lowest. According to the interquartile range of each gene Ct value, the three least variable reference genes were *β-actin*, *B2M*, and 1*8S rRNA* in the liver; *β-actin*, *EF1a*, and *GAPDH* in the foregut; *18S rRNA*, *B2M*, and *HPRT1* in the midgut; and *β-actin*, *GAPDH*, and *HPRT1* in the hindgut. In the foregut, midgut, and hindgut, the ranking of the expression levels of these six genes from highest to lowest was as follows: *18S rRNA* > *β-actin* > *EF1a* > *B2M* > *GAPDH* > *HPRT1*. In the liver, *18S rRNA* > *GAPDH* > *EF1a* > *β-actin* > *B2M* > *HPRT1*.

**Figure 2:**
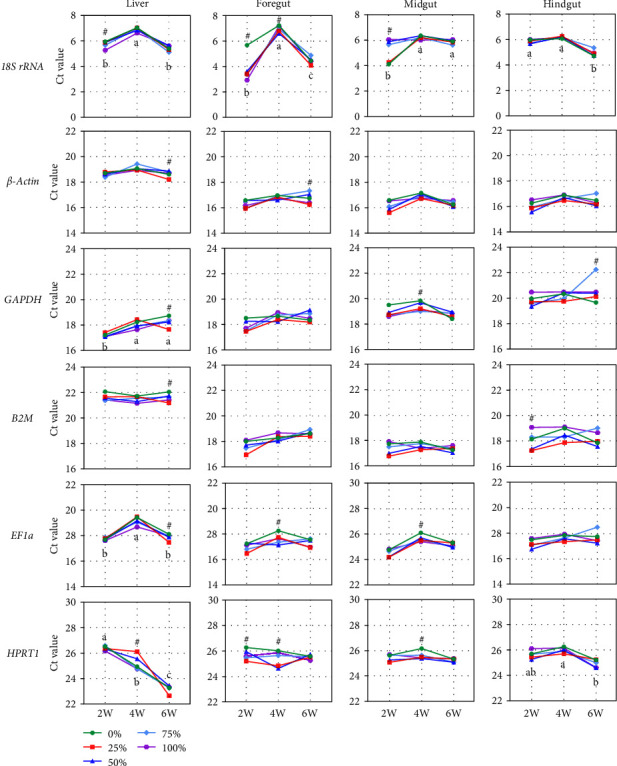
Changes in gene expression of reference genes in four tissues as a function of sampling time for each group (mean ± SD). The effect of different sampling time points were analyzed by applying analysis of variance (ANOVA) to the Ct values of the 0% group, followed by Dunn's test. Different alphabets indicate significant differences across sampling time points (*P* < 0.05). The effects of different soybean levels within each sampling time point were also analyzed by the same analytical method as above on the Ct values. ^#^Significant differences among different soybean meal levels (*P* < 0.05).

When gene expression data were visualized as a function of sampling time points or SBM levels, all reference genes had more unstable liver Ct values than those of the foregut, midgut, and hindgut. Using the Ct values of the livers sampled from the 0% group as an example, the Ct values of the reference genes were all variable across different sampling time points, except for *β-actin* and *B2M*, as shown in Figure 2. The Ct values of *18S rRNA* revealed more variables among different sampling time points in both of the four tissues. Similar findings were observed in other groups (Table S2). The variance analysis of the Ct values of the same tissue sampled at the same time point among different SBM levels showed that the Ct values of these reference genes were also affected by SBM levels, as shown in Figure 2.

### 3.3. Expression Stability of Candidate Reference Genes at Various SBM Levels

To confirm the effect of SBM levels on the expression stability of candidate reference genes, we inputted all the Ct values of samples from the same tissue sampled simultaneously into the programs mentioned above. Then received the ranks of the expression stability.

As shown in Tables S3–S6, the results showed that the stability rankings of these reference genes among different tissues sampled at the same time or the same tissue sampled at different sampling time points were varied. However, the top three stable reference genes provided by NormFinder and Delta Ct were unanimous, except for subtle differences in ranking (Tables S4 and S6). Compared with Bestkeeper, the top three stable reference genes provided by Genorm were more consistent with those provided by NormFinder and Delta Ct, with only a few discrepancies in the third gene.

The most unstable reference genes for each tissue sampled simultaneously, as provided by GeNorm, NormFinder, and Delta Ct, were consistent. The most unstable reference genes in the foregut and midgut were the same as those provided by BestKeeper. Meanwhile, *18S rRNA* had the highest frequency of occurrence for all the most unstable genes.

As shown in Table 2, comprehensive analysis rankings revealed that the top three stable reference genes provided only had minor differences when compared to those provided by GeNorm, NormFinder, or Delta Ct. *β-actin*, *HPRT1*, and *EF1a* were the top three stable reference genes of the foregut and midgut, except for *EF1a*, which was replaced by *B2M* in the 6 W midgut. In the hindgut, *β-actin* and *HPRT1* were ranked as the top two stable reference genes. Only *GAPDH* was the fixed gene ranked as the second most stable gene in the liver tissue at each sampling time point.

**Figure 3:**
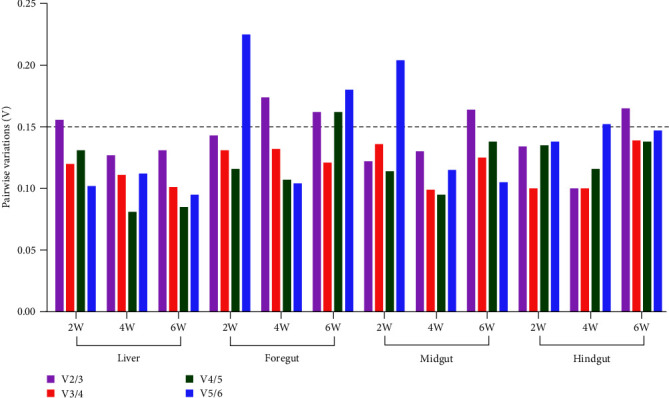
Optimal number of candidate reference genes among different soybean meal levels in hybrid yellow catfish. GeNorm was used for calculating the pairwise variation (Vn/Vn + 1, “*n*” means the number of reference genes).

In addition, V values of V_2/3_ in the liver sampled at 2 W, foregut sampled at 4 and 6 W, and midgut and hindgut sampled at 6 W all surpassed 0.15, indicating that three or more reference genes were required to normalize the target genes in RT-qPCR. For the remaining samples, V values of V_2/3_ were all less than 0.15, and two reference genes were suitable for normalizing target genes (Figure 3).

### 3.4. Expression Stability of Candidate Reference Genes among Different Tissues

To determine the expression stability of candidate reference genes among different tissues, we used Ct values from all tissues sampled at the same point of sampling time in each group. Then we ranked the expression stability of the reference genes.

In addition to slight differences in rankings, the top three stable reference genes provided by NormFinder and Delta Ct were consistent (Tables S8 and S10). They only had a subtle difference from those provided by GeNorm (Table S9) and by comprehensive analysis (Table 3). The top three stable reference genes of tissues sampled at 2 W, provided by all analytical programs except for BestKeeper, were *HPRT1*, *EF1a*, and *β-actin* in each group. While in samples of 4 W, *HPRT1* of 0% and 100% SBM groups, *β-actin* in the 75% SBM group was substituted with *18S rRNA*. In samples of 6 W, more differences in the top three stable genes were observed between the analytical programs or groups. According to the NormFinder and Delta Ct analyses, the top three stable reference genes in the 50% and 75% SBM groups were *EF1a*, *β-actin*, and *18S rRNA*, respectively. At the same time, they were *GAPDH*, *HPRT1*, and *EF1a* in the 50% SBM group and *β-actin*, *B2M*, and *EF1a* in the 75% SBM group by GeNorm analysis. Compared with NormFinder and Delta Ct analyses, the different data provided by comprehensive analysis only occurred in the 50% SBM group, in which *EF1a*, *GAPDH*, and *β-actin* were recommended as the top three most stable genes. The least stable genes in each group at different sampling times provided by the above four analytical programs were all focused on *GAPDH* or *B2M*.

The top three stable reference genes provided by Bestkeeper (Table S7) were vastly different from those provided by the four analytical programs above. However, no discernible pattern was found between sampling times or SBM levels. The relatively consistent thing is that the most stable reference genes were *HPRT1* at 2 W and *18S rRNA* at 6 W, and *B2M* was the least stable one at each sampling time point in all groups.

**Figure 4:**
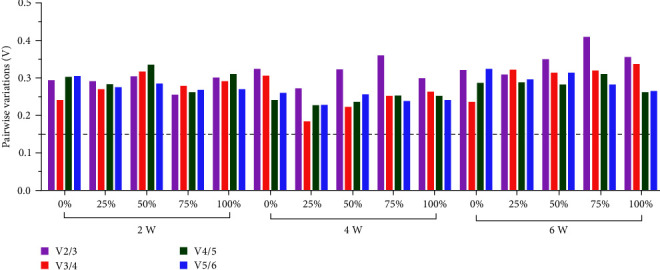
Optimal number of candidate reference genes among different tissues of hybrid yellow catfish. GeNorm was used for calculating the pairwise variation (Vn/Vn + 1, “*n*” means the number of reference genes).

As shown in Figure 4, the V values of V_2/3_, V_3/4_, V_4/5_, and V_5/6_ provided by GeNorm were all higher than 1.5, indicating more variable expression stability of these six reference genes across tissues and that more reference genes were needed when normalizing the target genes.

### 3.5. Expression Stability of Candidate Reference Genes among Different Points of Sampling Time

To determine the expression stability of candidate reference genes among different sampling time points, we input all Ct values of each tissue sampled at different sampling time points in each group and then obtain the expression stability rankings of these reference genes.

The top three stable reference genes provided by these analytical programs varied greatly, regardless of whether they were simultaneously sampled from different tissues or groups within the same tissue. In the liver, the top three stable reference genes identified by GeNorm (Table S13) were *β-actin*, *B2M*, and *GAPDH*. Using BestKeeper (Table S11), NormFinder (Table S12), Delta Ct (Table S14), and comprehensive analysis (Table 4), *β-actin*, *B2M*, and *18S rRNA* were identified in the 0%–50% SBM groups. In contrast, the results were consistent between BestKeeper and comprehensive analysis and between NormFinder and Delta Ct in the 75% and 100% SBM groups. The least stable reference gene was *HPRT1* in all programs. In the foregut, *β-actin* and *EF1a* were two of the top three reference genes identified using the above analytical programs. In addition, different programs may recommend *HPRT1*, *GAPDH*, or *B2M* in different groups. *β-actin* could be a suitable reference gene in the midgut and hindgut, with the highest frequency among the top three stable genes provided by the above programs in each group. The other two significantly depended on the analytical programs or the SBM levels. Except for *GAPDH*, *B2M*, and *EF1a*, which were occasionally considered the most unstable genes in the midgut or hindgut by BestKeeper or comprehensive analysis in some groups, *18S rRNA* was the least stable in the intestine provided by all the programs.

**Figure 5:**
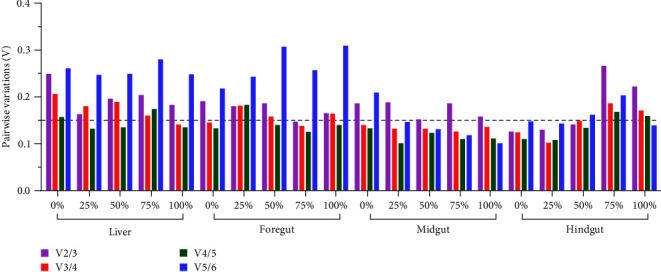
Optimal number of candidate reference genes among different sampling time points in each tissue of hybrid yellow catfish. GeNorm was used for calculating the pairwise variation (Vn/Vn + 1, “*n*” means the number of reference genes).

According to Figure 5, four-fifths of the V values of V_2/3_, more than half of the V values of V_3/4_, and a quarter of the V values of V_4/5_ were more significant than 0.15. The results indicated that three or four reference genes were required for RT-qPCR when compared at different sampling time points.

### 3.6. Evaluation and Validation of Screening Reference Genes

**Figure 6:**
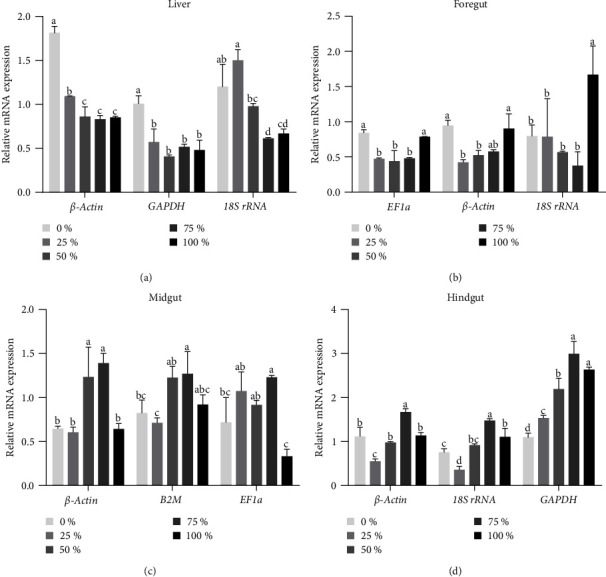
Changes in *MLKL* expression of samples sampled at 6 W normalized by the two most stable reference genes and the most unstable reference gene provided in Table 2.

The top two stable and most unstable reference genes, as shown in Table 2, were used to normalize *MLKL* expression levels in tissues sampled at 6 W, as shown in Figure 6. In the liver, the transcript levels of the *MLKL* gene significantly decreased in the 25%–100% SBM group compared to the 0% SBM group when normalized using *β-actin* and *GAPDH*, respectively (*P* < 0.05). However, a different tendency was observed when *18S rRNA* was used for normalization. When *EF1a* and *β-actin* were used as normalizer genes in the foregut, the transcript levels of *MLKL* in the 0% and 100% SBM groups were significantly higher than those in the 25%–50% SBM groups (*P* < 0.05). No significant differences were found between the 0% and 25%–50% SBM groups when *18S rRNA* was used as the normalizer (*P* < 0.05). However, when *β-actin* and *B2M* were used as normalizers in the midgut, the *MLKL* expression level in the 25% SBM group significantly decreased to the lowest level (*P* < 0.05). Simultaneously, the reverse result was obtained by normalizing to *EF1a*, the least stable gene. When *β-actin* and *18S rRNA* were used as normalizers of the hindgut, the expression levels of *MLKL* were significantly decreased to the lowest level in the 25% SBM group. They then significantly increased to the highest level in the 75% SBM group (*P* < 0.05). In contrast, it was significantly increased with increasing SBM levels and reached the highest level in the 75% and 100% SBM groups when normalized using *GAPDH* (*P* < 0.05).

## 4. Discussion

The present study showed greater consistency in the top three stable reference genes provided by NormFinder, GeNorm, Delta Ct, and the comprehensive analysis. The most stable genes obtained from one program were often the same or were among the top three stable genes obtained from the other programs. For example, when assessing the effects of different SBM levels on the stability ranking of reference genes in the foregut sampled at 2 W, the top three stable reference genes provided by Delta Ct were *EF1a* > *HPRT1* > *β-actin*, they were *HPRT1* > *EF1a* > *β-actin* by NormFinder or *β-actin/HPRT1* > *EF1a* by GeNorm. In contrast, the top three stable reference genes provided by Bestkeeper showed more significant differences from those provided by the above three analytical programs. Furthermore, a similar discrepancy existed between Bestkeeper and NormFinder, Delta Ct, Genorm, and comprehensive analysis when the least unstable reference gene was analyzed. Several studies have mentioned different analytical programs that result in different stability rankings [20, 31]. This disparity was caused by differences in the algorithms and procedures used by each computational program.

RT-qPCR is a popular molecular biology technique for studying the mechanisms of the nutritional hazards caused by plant protein-based diets [9, 10, 13, 17]. However, it is yet to be determined whether the expression stability of reference genes has been tested in plant protein-based diet experiments. In the present study, six candidate genes that cover multiple physiological activities of cells involved in cytoskeleton formation (*β-actin*), protein synthesis (*EF1a*), glycolysis canalization (*GAPDH*), purine nucleotide cycle (*HPRT1*), protein translation (*18S rRNA*), immune response, and structural protein (*B2M*) were chosen based on previous studies on fish [4, 28, 31]. The expression variations in different tissues reflected by violin maps or the interquartile range of Ct values of each gene indicated the tissue-dependent expression of these reference genes in hybrid yellow catfish fed with different SBM levels. The more unstable Ct values in the liver compared to the foregut, midgut, or hindgut, reflected by the visualizing data of gene expression as a function of different sampling time points or SBM levels in each tissue, was another support for this organ-specific feature. The optimal combination obtained from GeNorm, represented by V values of V_n_/V_n + 1_ (V values of V_2/3_, V_3/4_, V_4/5_, and V_5/6_ were all greater than 1.5) (Figure 4), also indicating that the expression stability of these reference genes was more variable in different tissues. Therefore, when tissue factors are considered, more reference genes should be chosen to normalize the target genes.

When the factors of SBM levels or different sampling time points were evaluated, significant discrepancies in the ranking were observed among the same tissue sampled at different sampling time points or among different SBM levels in the same tissue. The stability rankings also varied among the tissues sampled simultaneously (Tables 2 and 4). For example, the top three stable reference genes in different tissues sampled at 2 W were nearly consistent with each other at different SBM levels. At the same sampling time, there were no regular rules for 4 and 6 W among the SBM levels (Table 3). *GAPDH*, the least stable reference gene in the tissues of each SBM level sampled at 2 W, was ranked among the top three stable genes in the tissues of the 0% and 50% SBM levels sampled at 6 W. These results revealed that the expression stability of the six reference genes in hybrid yellow catfish varied based on the tissues, sampling time points, and SBM dosage. Many studies have reported that experimental conditions, such as dietary restriction [32], metals [22], viral infection [33, 34], and herbicides [20], influence the expression of reference genes that have traditionally been considered as reference genes (e.g., *GAPDH*, *HPRT1*, and *18S rRNA*). This could be attributed to each organ's different functions and heterogeneity and the characteristic reactions of the evaluated reference genes to specific conditions [35].

Among the six candidate reference genes assessed in this study, *18S rRNA*, a part of ribosomal RNA, showed the most abundant expression (Ct values ranged from 2.93 to 7.04). For the most of specific target genes measured in typical RT-qPCR experiments, they are often expressed at relatively low levels. Hence, a significant difference in the expression of *18S rRNA* and its target genes will reduce the accuracy of RT-qPCR; minor changes in target gene expression between different groups are challenging to observe. From this point of view, *18S rRNA* is unsuitable as a control gene for normalizing low-copy target genes. In studies on rainbow trout (*Oncorhynchus mykiss*) [22], zebrafish (*Danio rerio*) [20], and grass carp [36], *18S rRNA* was excluded from the candidate reference genes. In theory, as a ribosomal RNA, *18S rRNA* expression is hardly affected by conditions that influence the expression of mRNA [33, 36], whereas its expression is indeed influenced by experimental conditions [37]. In Prenant's schizothoracin (*Schizothorax prenanti*), challenged by the pathogen, *18S rRNA* was also considered an unsuitable reference gene both *in vitro* and *in vivo* when compared among different treatments [21]. In our study, although *18S rRNA* was ranked among the top three stable reference genes by all the analytical programs when the factors of SBM levels, tissues, and sampling time points were integrated, it was often considered the least stable when a single factor was considered. In conclusion, *18S rRNA* may not be an appropriate reference gene for studies on plant protein-based diet-induced nutritional hazards in hybrid yellow catfish.

The other five candidate reference genes used in our study are widely used for RT-qPCR in fish studies [33, 38]. In the present study, *GAPDH* and *B2M* were considered the two most unstable reference genes when the different tissues were the dependent variables. When the different SBM levels or sampling time points were considered as the dependent variables, *GAPDH* and *B2M* were also ranked out of the top three stable genes in most rankings. In fish, it has been reported that the expression of *GAPDH* and *B2M* varies with tissues and developmental stages [39], exposure to harmful substances [20], the severity of oxidative stress, and inflammatory states [40–43]. Hybrid yellow catfish is an SBM intolerance species; soybean antigen protein, which accounts for approximately 40% of total SBM protein, frequently induces oxidative stress and cell apoptosis, ultimately causing damage to the morphological structure of the intestine [44]. Replacing a fish meal with plant protein sources often causes intestinal inflammation and growth inhibition. Soybean protein, a fishmeal replacement protein, has been associated with intestinal damage. Genes involved in intestinal barrier development, including the physical, chemical, and immunological barriers, frequently show dysregulated expression [13, 24]. In our study, the unstable *GAPDH* and *B2M* expression may result from oxidative stress or inflammation caused by SBM. Therefore, *GAPDH* and *B2M* are not recommended as internal reference genes when using RT-qPCR to normalize target genes in nutrition research related to SBM.

*HPRT1*, a coding gene encoding hypoxanthine-guanine phosphoribosyl transferase (HGPRT) involved in the purine nucleotide cycle, exhibits species- [31, 39], tissue-, and test substance-dependent expression [20, 22]. Similar findings were found in the current study. For example, compared with the intestine, the Ct values of *HPRT1* in the liver demonstrated more variability across different sampling time points at each SBM level, and *HPRT1* has been deemed the least stable reference gene in all programs. In addition, *HPRT1* was ranked out of the top three stable genes by all programs except Bestkeeper when the stability ranking of these candidate genes was integrated with tissue factors, sampling time points, and SBM levels. Although the stability ranking of *HPRT1* showed different expressions regardless of tissue or group, *HRPT1* still has value as a reference gene for the intestine when points of sampling time or SBM levels are considered. Therefore, we do not recommend using *HPRT1* as a reference gene for the liver of hybrid yellow catfish.

*β-Actin* has often been reported as the most stable reference gene, less impacted by factors of tissues, developmental stages, or stress conditions in both fish and mammals. Pacific abalone (*abalone Haliotis discus hannai*) [45], rainbow trout [46], half-smooth tongue sole (*Cynoglossus semilaevis*) [47], zebrafish [48], and *β-actin* have all been reported to be suitable reference genes. However, in normal grass carp and Jian carp (*Cyprinus carpio var. jian*), *β-actin* was unsuitable as a reference gene when analyzed in different tissues [36, 49]. Our study consistently ranked *β-actin* among the top three stable reference genes, regardless of the SBM levels, sampling time points, or tissues. In our study, the expression of *EF1a* was time- and dose-dependent with SBM levels, as evidenced by the different rankings between different SBM levels in the same tissue or between different sampling time points in the same tissue. It was ranked among the top three stable genes when evaluated in different tissues or when all the Ct values were combined. Similar findings have been reported in different tissues of adult Atlantic salmon (*Atlantic salmon*) [50] and zebrafish [51]. These findings suggest that *EF1a* is a suitable reference gene for hybrid yellow catfish when different tissues are used as variables.

According to the V values of V_n_/V_n + 1_ provided by GeNorm, most of the V values of V_2_/V_3_ were less than 0.15 when ranking the stability among different SBM levels, and all of the V values were larger than 0.15 when ranking the stability among different tissues. Furthermore, half of the V values of V_3_/V_4_ were less than 0.15 when ranking the stability among different sampling time points. These findings showed that the expression stability of the reference genes varied more between tissues than between SBM levels and sampling time points. Therefore, a suitable number of reference genes for target genes should be determined for specific experimental purposes in hybrid yellow catfish fed a soybean meal-based diet. Moreover, using multiple genes as controls for normalization may be a suitable theoretical approach. On the other hand, increasing the number of reference genes increases experimental complexity and cost. Further, multiple reference genes can also lead to instability [52]. Thus, suitable reference genes that balance cost and precision are required.

When the screening of reference genes was evaluated and validated by normalizing the relative expression of *MLKL* samples sampled at 6 W, the tendencies of *MLKL* transcript levels among different SBM levels were almost consistent when the two stable genes were used for calibration. However, a different trend was observed when the least stable gene was used for calibration. These results confirm the stability of the selected reference genes.

## 5. Conclusion

In our study, *β-actin* and *GAPDH* in the liver and *β-actin*, *HPRT1*, and *EF1a* in the intestine were the most stable reference genes when SBM levels were considered. When different tissue factors were considered, *HPRT1* and *EF1a* for 2 W and *EF1a* and *β-actin* for 4 and 6 W were proposed as the two stable reference genes. When the sampling time points were considered, *β-actin*, *EF1a*, and *HPRT1* were the top three stable reference genes in the intestine, whereas *β-actin* and *B2M* were the two most stable reference genes in the liver. The present study demonstrated that dietary SBM levels significantly affected the expression stability of reference genes in hybrid yellow catfish. The stability was time-, dose-, and tissue-dependent. The results also revealed that the expression stability of the reference genes varied more between tissues than between SBM levels and sampling time points. Therefore, we strongly recommend screening for stable reference genes in hybrid yellow catfish fed a plant protein-based diet before performing RT-qPCR experiments to acquire more accurate and reliable data.

## Figures and Tables

**Table 1:** Information of the six candiated reference genes.

Abbreviation	Reference genes name	Accession no.	Primer sequence (5′−3′)	Amplicon size (bp)	Correlation coefficients (R2)	Amplification efficiency (%)
*18S rRNA*	18S ribosomal RNA	XR_003445016	F: GGCCGTTCTTAGTTGGTGGA	179	0.9973	95.21
			R: CCCGGACATCTAAGGGCATC			
*β-Actin*	Beta-actin	XM_027148463	F: CCTTGACTTCGAGCAGGAGA	115	0.9970	110.98
			R: GGGACACCTGAACCTCTCATT			
*GAPDH*	Glyceraldehyde 3-phosphate dehydrogenase	KP938521	F: AGCGAGAGAGACCCAGCTAA	127	0.9960	110.20
			R: TGACTCTCTTGGCACCTCCT			
*EF1a*	Elongation factor 1-alpha	XM_027175544	F: CTACAACCCTGCTGCCGTT	144	0.9972	107.90
			R: TCCAGGAGAGTAGTGCCGC			
*B2M*	Beta-2-microglobulin	KP938520	F: GCCGTAACCCTGGCGTATTT	129	0.9967	113.08
			R: GGTCGGTCTGCTTGGCATTA			
*HPRT1*	hypoxanthine phosphoribosyltransferase 1	XM_027136099	F: AATCATGGACAGGACTGAGCG	149	0.9827	114.23
			R: GATCGCTGTTGCGGTTCAG			
*MLKL*	mixed lineage kinase domain-like	XM_027135117	F: CAGACCTGAGGAGCTGACCT	144	0.9909	112.81
			R: AGTTGATTTGGGCTGGTGCT			

**Table 2:** Stability analysis of candidate reference genes at various soybean meal levels (comprehensive analysis).

Tissues	Rank	Sampling times							
		2 W			4 W			6 W	
		Genes	Arithmetic mean value		Genes	Arithmetic mean value		Genes	Arithmetic mean value
Liver	1	*β*-Actin	1.19		18S rRNA	1.41		*β*-Actin	1.57
	2	GAPDH	1.86		GAPDH	1.86		GAPDH	2.00
	3	18S rRNA	3.34		EF1a	2.06		B2M	2.28
	4	B2M	3.66		B2M	4.23		EF1a	3.34
	5	EF1a	4.12		*β*-Actin	4.40		HPRT1	4.43
	6	HPRT1	5.42		HPRT1	6.00		18S rRNA	5.73

Foregut	1	HPRT1	1.41		*β*-Actin	1.19		EF1a	1.19
	2	*β*-Actin	1.73		18S rRNA	1.41		*β*-Actin	1.41
	3	EF1a	2.06		EF1a	3.46		HPRT1	3.46
	4	B2M	4.23		GAPDH	3.66		GAPDH	3.46
	5	GAPDH	4.73		HPRT1	4.73		B2M	5.00
	6	18S rRNA	6.00		B2M	6.00		18S rRNA	6.00

Midgut	1	*β*-Actin	1.73		EF1a	1.19		*β*-Actin	1.32
	2	HPRT1	1.86		*β*-Actin	1.41		B2M	2.00
	3	EF1a	2.21		HPRT1	3.00		HPRT1	2.71
	4	GAPDH	2.99		B2M	4.00		18S rRNA	3.16
	5	B2M	4.73		GAPDH	5.00		GAPDH	4.47
	6	18S rRNA	6.00		18S rRNA	6.00		EF1a	6.00

Hindgut	1	*β*-Actin	1.19		HPRT1	1.86		*β*-Actin	1.19
	2	HPRT1	1.86		EF1a	1.86		18S rRNA	1.86
	3	GAPDH	3.22		*β*-Actin	2.06		HPRT1	2.78
	4	18S rRNA	3.83		18S rRNA	3.83		EF1a	3.66
	5	EF1a	4.23		GAPDH	4.23		B2M	4.47
	6	B2M	5.23		B2M	5.23		GAPDH	6.00

**Table 3:** Stability analysis of candidate reference genes among different tissues (comprehensive analysis).

Sampling time points	Rank	Soybean meal levels										
		0%		25%		50%		75%		100%		
		Genes	Arithmetic mean value	Genes	Arithmetic mean value	Genes	Arithmetic mean value	Genes	Arithmetic mean value	Genes	Arithmetic mean value	
2W	1	HPRT1	1.19	HPRT1	1.19	EF1a	1.19	HPRT1	1.00	HPRT1	1.00	
	2	EF1a	1.57	EF1a	1.41	HPRT1	1.41	EF1a	1.68	EF1a	1.68	
	3	*β*-Actin	3.22	*β*-Actin	3.22	*β*-Actin	3.41	*β*-Actin	3.22	*β*-Actin	3.00	
	4	18S rRNA	3.36	18S rRNA	4.23	18S rRNA	4.47	18S rRNA	3.94	18S rRNA	4.47	
	5	B2M	5.23	GAPDH	4.82	B2M	4.68	B2M	4.95	B2M	4.68	
	6	GAPDH	5.73	B2M	5.48	GAPDH	5.05	GAPDH	5.73	GAPDH	5.73	

4W	1	EF1a	1.32	EF1a	1.50	EF1a	1.19	EF1a	1.86	EF1a	1.19	
	2	*β*-Actin	2.11	*β*-Actin	2.00	*β*-Actin	2.21	18S rRNA	1.86	18S rRNA	1.86	
	3	18S rRNA	2.28	HPRT1	2.71	HPRT1	2.71	HPRT1	2.38	*β*-Actin	2.63	
	4	HPRT1	3.36	18S rRNA	2.83	18S rRNA	2.83	GAPDH	3.16	HPRT1	3.66	
	5	GAPDH	4.73	GAPDH	4.40	B2M	5.23	*β*-Actin	3.87	B2M	4.95	
	6	B2M	6.00	B2M	6.00	GAPDH	5.73	B2M	6.00	GAPDH	5.73	

6W	1	EF1a	1.19	EF1a	1.00	EF1a	1.32	EF1a	1.57	EF1a	1.00	
	2	GAPDH	1.57	18S rRNA	1.68	GAPDH	2.38	*β*-Actin	2.21	*β*-Actin	2.11	
	3	*β*-Actin	2.91	*β*-Actin	3.22	*β*-Actin	3.16	18S rRNA	2.21	18S rRNA	2.71	
	4	18S rRNA	3.72	GAPDH	3.72	HPRT1	3.16	B2M	3.50	GAPDH	4.00	
	5	HPRT1	5.00	HPRT1	5.00	18S rRNA	3.22	HPRT1	3.94	HPRT1	4.40	
	6	B2M	6.00	B2M	6.00	B2M	6.00	GAPDH	5.73	B2M	6.00	

**Table 4:** Stability analysis of candidate reference genes among different sampling time points (comprehensive analysis).

Tissues	Rank	Soybean meal levels										
		0%		25%		50%		75%		100%		
		Genes	Arithmetic mean value	Genes	Arithmetic Mean value	Genes	Arithmetic mean value	Genes	Arithmetic mean value	Genes	Arithmetic mean value	
Liver	1	*β*-Actin	1.00	*β*-Actin	1.19	*β*-Actin	1.00	*β*-Actin	1.19	*β*-Actin	1.32	
	2	B2M	1.68	B2M	1.73	B2M	2.06	B2M	2.06	B2M	1.41	
	3	18S rRNA	3.41	18S rRNA	2.63	18S rRNA	2.63	GAPDH	2.78	EF1a	2.91	
	4	EF1a	4.00	GAPDH	3.94	GAPDH	4.16	EF1a	3.13	GAPDH	3.94	
	5	GAPDH	4.40	EF1a	4.73	EF1a	4.47	18S rRNA	4.73	18S rRNA	4.73	
	6	HPRT1	6.00	HPRT1	6.00	HPRT1	6.00	HPRT1	6.00	HPRT1	6.00	

Foregut	1	*β*-Actin	1.00	*β*-Actin	1.00	*β*-Actin	1.00	EF1a	1.32	EF1a	1.19	
	2	HPRT1	2.21	GAPDH	2.06	EF1a	1.68	*β*-Actin	1.68	*β*-Actin	1.41	
	3	EF1a	2.91	EF1a	2.45	GAPDH	3.00	HPRT1	2.83	HPRT1	3.46	
	4	GAPDH	3.13	HPRT1	4.00	B2M	4.47	B2M	3.41	GAPDH	3.46	
	5	B2M	5.00	B2M	5.00	HPRT1	4.47	GAPDH	4.73	B2M	5.00	
	6	18S rRNA	6.00	18S rRNA	6.00	18S rRNA	6.00	18S rRNA	6.00	18S rRNA	6.00	

Midgut	1	*β*-Actin	1.19	*β*-Actin	1.32	B2M	1.19	*β*-Actin	1.00	*β*-Actin	1.32	
	2	HPRT1	1.41	EF1a	2.00	GAPDH	2.45	EF1a	2.11	HPRT1	2.38	
	3	GAPDH	3.72	HPRT1	2.71	*β*-Actin	2.78	B2M	3.00	EF1a	2.91	
	4	EF1a	3.87	GAPDH	3.16	18S rRNA	3.83	HPRT1	3.36	18S rRNA	3.83	
	5	B2M	4.16	B2M	4.68	HPRT1	3.94	GAPDH	4.73	GAPDH	4.16	
	6	18S rRNA	6.00	18S rRNA	5.73	EF1a	4.95	18S rRNA	6.00	B2M	4.16	

Hindgut	1	GAPDH	1.19	*β*-Actin	1.32	*β*-Actin	1.19	*β*-Actin	1.00	EF1a	1.41	
	2	*β*-Actin	1.73	HPRT1	2.21	EF1a	2.00	B2M	2.00	*β*-Actin	1.68	
	3	HPRT1	2.45	EF1a	2.59	B2M	2.99	HPRT1	3.46	HPRT1	3.41	
	4	EF1a	4.23	GAPDH	3.34	GAPDH	3.66	18S rRNA	3.76	18S rRNA	3.83	
	5	B2M	5.23	B2M	4.00	HPRT1	3.87	EF1a	3.87	GAPDH	3.94	
	6	18S rRNA	5.42	18S rRNA	6.00	18S rRNA	6.00	GAPDH	6.00	B2M	4.95	

## Data Availability

The data that support the findings of this study will be available on reasonable request from the corresponding author.

## Supplementary Materials

Supplementary Materials Table S1: Formulation and proximate composition of experimental diets. Table S2: changes in gene expression of six candidate reference genes among different sampling time points using the raw Ct values. Table S3: stability analysis of six candidate reference genes at various soybean meal levels (BestKeeper). Table S4: stability analysis of six candidate reference genes at various soybean meal levels (NormFinder). Table S5: stability analysis of six candidate reference genes at various soybean meal levels (GeNorm). Table S6: stability analysis of six candidate reference genes at various soybean meal levels (Delta Ct). Table S7: stability analysis of six candidate reference genes among different tissues (BestKeeper). Table S8: stability analysis of six candidate reference genes among different tissues (NormFinder). Table S9: stability analysis of six candidate reference genes among different tissues (GeNorm). Table S10: stability analysis of six candidate reference genes among different tissues (Delta Ct). Table S11: stability analysis of six candidate reference genes at different sampling time points (BestKeeper). Table S12: stability analysis of six candidate reference genes at different sampling time points (NormFinder). Table S13: stability analysis of six candidate reference genes in different sampling time points (GeNorm). Table S14: stability analysis of six candidate reference genes at different sampling time points (Delta Ct). Figure S1: melting curves of six candidate reference genes.
