# Supplementary material for: Screening and Stability Analysis of Reference Genes for Gene Expression Normalization in Hybrid Yellow Catfish (Pelteobagrus fulvidraco ♀ × Pelteobagrus vachelli ♂) Fed Diets Containing Different Soybean Meal Levels
Source: Aquac Nutr. 2023 Sep 22;2023:1232518. doi: 10.1155/2023/1232518 (PMC10541299; doi:10.1155/2023/1232518)
Supplement: Supplementary Materials — Table S1: Formulation and proximate composition of experimental diets. Table S2: changes in gene expression of six candidate reference genes among different sampling time points using the raw Ct values. Table S3: stability analysis of six candidate reference genes at various soybean meal levels (BestKeeper). Table S4: stability analysis of six candidate reference genes at various soybean meal levels (NormFinder). Table S5: stability analysis of six candidate reference genes at various soybean meal levels (GeNorm). Table S6: stability analysis of six candidate reference genes at various soybean meal levels (Delta Ct). Table S7: stability analysis of six candidate reference genes among different tissues (BestKeeper). Table S8: stability analysis of six candidate reference genes among different tissues (NormFinder). Table S9: stability analysis of six candidate reference genes among different tissues (GeNorm). Table S10: stability analysis of six candidate reference genes among different tissues (Delta Ct). Table S11: stability analysis of six candidate reference genes at different sampling time points (BestKeeper). Table S12: stability analysis of six candidate reference genes at different sampling time points (NormFinder). Table S13: stability analysis of six candidate reference genes in different sampling time points (GeNorm). Table S14: stability analysis of six candidate reference genes at different sampling time points (Delta Ct). Figure S1: melting curves of six candidate reference genes. [file 1232518.f1.docx]

**Table S1.** Formulation and proximate composition of experimental diets (Dry matter basis, g/kg).

| Ingredients ^a^ | 0% | 25% | 50% | 75% | 100% |
| --- | --- | --- | --- | --- | --- |
| Fish meal | 400 | 300 | 200 | 100 | 0 |
| Soybean meal | 0 | 100 | 200 | 300 | 400 |
| Vital gluten | 120 | 120 | 120 | 120 | 120 |
| Whole wheat flour | 351 | 303 | 251 | 205 | 155 |
| Soy protein concentrate | 0 | 38 | 80 | 120 | 160 |
| Squid powder | 15 | 15 | 15 | 15 | 15 |
| Fish oil | 32 | 37 | 42 | 45 | 50 |
| Soybean oil | 32 | 37 | 42 | 45 | 50 |
| Choline chloride | 20 | 20 | 20 | 20 | 20 |
| Premix of vitamins and mineral salts ^b^ | 10 | 10 | 10 | 10 | 10 |
| Ca(H**_2_**PO_4_)_2_ | 20 | 20 | 20 | 20 | 20 |
| total | 1000 | 1000 | 1000 | 1000 | 1000 |
| Analyzed nutrients compositions (Dry matter basis, g/kg) | | | | | |
| Moisture | 40.4 | 39.1 | 38.4 | 39.2 | 38.9 |
| Crude lipid | 104.3 | 100.7 | 101.0 | 100.2 | 100.5 |
| Crude protein | 440.5 | 444.8 | 445.1 | 440.8 | 440.5 |

^a^ All the ingredients were supplied by Guangdong Liankun Co., Ltd.

^b^ Vitamin and mineral salt premix (mg/kg premix): vitamin A, 115.5; vitamin D_3_, 3; vitamin E, 4400; vitamin K_3_, 1200; vitamin B_1_, 660; vitamin B_2_, 1950; vitamin B_6_, 880; vitamin B_12_, 4.8; vitamin C, 11500; calcium D-pantothenate, 2750; niacinamide, 3300; folic acid, 275; D-biotin, 11; inositol, 5000; magnesium, 5000; zinc, 1900; manganese, 1250; copper, 830; iron, 4100; cobalt, 160; iodine, 110; selenium, 32.

**Table S2.** Changes in genes expression of six candidate reference genes among different sampling time points using the raw Ct values (mean ± SD)

|  |  | Average Ct value ± SD | | | | | | | | | | | |
| --- | --- | --- | --- | --- | --- | --- | --- | --- | --- | --- | --- | --- | --- |
|  |  | Liver | | | Foregut | | | Midgut | | | Hindgut | | |
| Genes | Groups | 2W | 4W | 6W | 2W | 4W | 6W | 2W | 4W | 6W | 2W | 4W | 6W |
| 18S rRNA | 0% | 5.95±0.28^b^ | 7.04±0.56^a^ | 5.39±0.42^b^ | 5.68±0.66^b^ | 7.24±0.35^a^ | 4.46±1.01^c^ | 4.12±1.69^b^ | 6.38±0.77^a^ | 5.93±0.43^a^ | 6.00±0.24^a^ | 6.08±0.57^a^ | 4.72±0.52^b^ |
|  | 25% | 5.91±0.33^b^ | 7.03±0.23^a^ | 5.32±0.34^c^ | 3.42±1.69^b^ | 6.80±0.24^a^ | 4.10±1.05^b^ | 4.27±1.62^b^ | 6.22±0.47^a^ | 5.88±0.37^a^ | 5.89±0.34^a^ | 6.28±0.62^a^ | 4.90±0.71^b^ |
|  | 50% | 5.89±0.31^b^ | 6.83±0.71^a^ | 5.64±0.78^b^ | 3.62±1.85^b^ | 6.61±0.21^a^ | 4.54±1.41^b^ | 5.90±0.40 | 6.37±0.58 | 6.00±0.51 | 5.68±0.73^ab^ | 6.24±0.60^a^ | 4.74±0.91^b^ |
|  | 75% | 5.67±0.47^b^ | 6.96±0.51^a^ | 5.12±0.353^b^ | 3.38±1.56^b^ | 6.94±0.46^a^ | 4.89±1.20^b^ | 5.67±0.61 | 6.23±0.78 | 5.64±0.43 | 5.74±0.35 | 6.18±0.53 | 5.36±1.18 |
|  | 100% | 5.27±0.22^b^ | 6.61±0.46^a^ | 5.61±0.65^b^ | 2.93±1.72^b^ | 7.17±0.33^a^ | 4.42±1.16^b^ | 6.08±0.19 | 6.03±0.97 | 6.09±0.42 | 6.03±0.36^a^ | 6.18±0.78^a^ | 4.96±0.80^b^ |
| β-actin | 0% | 18.70±0.22 | 19.07±1.22 | 18.61±0.35 | 16.57±0.85 | 16.96±0.65 | 16.73±0.58 | 16.57±1.50 | 17.14±0.50 | 16.24±0.76 | 16.25±0.46 | 16.86±0.72 | 16.46±0.50 |
|  | 25% | 18.80±0.98 | 18.94±0.61 | 18.20±0.42 | 15.93±0.74 | 16.83±0.44 | 16.24±0.41 | 15.61±1.11^b^ | 16.71±0.27^a^ | 16.20±1.05^b^ | 15.87±0.55 | 16.45±0.44 | 16.17±0.75 |
|  | 50% | 18.63±0.34 | 19.05±0.85 | 18.87±0.42 | 16.57±0.47 | 16.59±0.46 | 17.03±0.74 | 15.86±0.56^b^ | 17.07±0.31^a^ | 16.08±0.81^b^ | 15.55±0.23^b^ | 16.68±0.67^a^ | 16.03±0.72^ab^ |
|  | 75% | 18.38±0.17^b^ | 19.42±0.30^a^ | 18.78±0.40^b^ | 15.94±0.63^b^ | 16.91±0.46^a^ | 17.32±0.77^a^ | 16.08±0.44^b^ | 16.90±0.16^a^ | 16.45±0.19^b^ | 15.93±0.48^b^ | 16.58±0.31^ab^ | 17.00±0.97^a^ |
|  | 100% | 18.53±0.77 | 18.93±0.70 | 18.70±0.34 | 16.16±0.62 | 16.71±0.47 | 16.38±0.70 | 16.53±0.49 | 16.73±0.63 | 16.55±0.92 | 16.49±1.12 | 16.87±0.70 | 16.25±0.66 |
| GAPDH | 0% | 17.22±0.48^b^ | 18.24±0.96^a^ | 18.74±0.32^a^ | 18.51±1.17 | 18.66±0.85 | 18.36±0.57 | 19.54±1.45 | 19.87±0.38 | 18.43±0.99 | 19.91±0.42 | 20.27±0.90 | 19.61±0.67 |
|  | 25% | 17.43±0.94 | 18.43±0.90 | 17.65±0.71 | 17.46±0.79 | 18.40±0.84 | 18.19±0.15 | 18.75±0.90 | 19.24±0.52 | 18.61±0.62 | 19.67±0.37 | 19.70±0.25 | 20.07±0.86 |
|  | 50% | 17.09±0.82^b^ | 17.94±0.74^ab^ | 18.24±0.23^a^ | 18.27±0.73 | 18.23±0.60 | 19.14±0.73 | 18.94±0.49 | 19.71±0.32 | 18.97±0.98 | 19.30±0.43^b^ | 20.35±0.81^a^ | 20.33±0.57^a^ |
|  | 75% | 17.10±0.26^b^ | 17.92±0.25^a^ | 18.35±0.44^a^ | 17.51±0.47^b^ | 18.75±0.91^a^ | 18.87±0.84^a^ | 18.74±0.61 | 19.03±0.43 | 18.91±0.87 | 19.54±0.74^b^ | 19.90±0.50^b^ | 22.16±1.59^a^ |
|  | 100% | 17.10±0.65^b^ | 17.64±0.54^b^ | 18.35±0.20^a^ | 17.71±1.00^b^ | 18.95±0.69^a^ | 18.48±0.69^ab^ | 18.63±0.47 | 19.12±0.85 | 18.87±1.17 | 20.41±1.27 | 20.42±1.13 | 20.42±1.63 |
| B2M | 0% | 22.07±0.44 | 21.73±1.40 | 22.05±0.25 | 18.00±1.00 | 18.27±0.76 | 18.62±1.00 | 17.74±2.03 | 17.89±0.73 | 17.25±0.65 | 18.13±0.54 | 18.99±1.52 | 17.85±0.64 |
|  | 25% | 21.66±0.98 | 21.68±0.52 | 21.19±0.75 | 16.94±0.87 | 18.36±1.41 | 18.40±1.28 | 16.76±1.70 | 17.28±0.56 | 17.38±0.88 | 17.25±0.66 | 17.87±0.59 | 17.97±0.88 |
|  | 50% | 21.56±0.66 | 21.29±0.88 | 21.73±0.36 | 17.70±0.43 | 18.02±1.00 | 18.68±1.21 | 16.99±0.43 | 17.54±0.35 | 17.03±1.00 | 17.37±0.31 | 18.45±1.13 | 17.57±0.65 |
|  | 75% | 21.38±0.93 | 21.55±0.38 | 21.66±0.34 | 17.46±0.99^b^ | 18.11±0.93^ab^ | 18.92±0.67^a^ | 17.50±0.71 | 17.77±0.58 | 17.40±0.43 | 18.30±0.64 | 18.31±0.79 | 19.01±1.32 |
|  | 100% | 21.42±0.83 | 21.15±0.68 | 21.42±0.17 | 18.08±1.24 | 18.66±0.74 | 18.59±1.74 | 17.92±0.84 | 17.38±0.54 | 17.61±0.98 | 19.07±1.93 | 19.09±1.49 | 18.64±1.29 |
| EF1a | 0% | 17.69±0.60^b^ | 19.42±0.67^a^ | 18.12±0.15^b^ | 17.24±0.82 | 18.26±0.74 | 17.55±0.80 | 16.77±1.63 | 18.11±0.47 | 17.33±1.08 | 17.53±1.00 | 17.88±0.93 | 17.78±0.89 |
|  | 25% | 17.76±1.07^b^ | 19.48±0.36^a^ | 17.48±0.33^b^ | 16.48±0.84^b^ | 17.74±0.93^a^ | 16.94±0.38^ab^ | 16.20±1.23 | 17.51±0.17 | 17.31±1.06 | 17.17±0.80 | 17.38±0.46 | 17.52±0.88 |
|  | 50% | 17.69±0.93^b^ | 19.16±0.93^a^ | 17.89±0.33^b^ | 17.28±0.68 | 17.14±0.58 | 17.50±0.71 | 16.21±0.77^b^ | 17.71±0.15^a^ | 16.97±1.35^ab^ | 16.77±0.44 | 17.61±0.62 | 17.26±0.99 |
|  | 75% | 17.85±0.95^b^ | 19.06±0.04^a^ | 17.98±0.54^b^ | 16.81±0.95 | 17.37±0.68 | 17.63±0.80 | 16.65±0.65^b^ | 17.58±0.24^a^ | 16.99±0.70^ab^ | 17.10±0.76 | 17.67±0.34 | 18.53±1.66 |
|  | 100% | 17.61±0.58^b^ | 18.68±0.46^a^ | 17.98±0.26^b^ | 17.13±0.80 | 17.64±0.52 | 16.98±0.74 | 16.82±0.94 | 17.41±0.71 | 17.08±1.37 | 17.64±1.45 | 17.98±0.84 | 17.46±1.01 |
| HPRT1 | 0% | 26.50±0.44^a^ | 24.93±1.07^b^ | 23.27±0.56^c^ | 26.28±0.41 | 26.02±0.79 | 25.59±0.89 | 25.62±1.22 | 26.17±0.23 | 25.33±0.61 | 25.75±0.56^ab^ | 26.32±0.77^a^ | 25.26±0.42^b^ |
|  | 25% | 26.34±0.97^a^ | 26.11±0.65^a^ | 22.65±0.69^b^ | 25.21±0.92 | 24.85±0.72 | 25.52±0.64 | 25.08±1.04 | 25.48±0.58 | 25.33±0.60 | 25.48±0.63 | 25.75±0.45 | 25.30±0.53 |
|  | 50% | 26.31±0.96^a^ | 25.55±1.27^a^ | 23.41±0.56^b^ | 25.95±0.56^a^ | 24.64±0.60^b^ | 25.74±0.75^a^ | 25.26±0.80 | 25.37±0.38 | 25.08±0.84 | 25.30±0.33^ab^ | 26.05±0.90^a^ | 24.64±0.63^b^ |
|  | 75% | 26.57±1.02^a^ | 24.77±0.50^b^ | 23.27±0.49^c^ | 25.47±0.46 | 25.65±0.81 | 25.56±0.78 | 25.63±0.47 | 25.64±0.25 | 25.08±0.66 | 25.72±0.78 | 25.95±0.41 | 25.03±1.14 |
|  | 100% | 26.16±0.31^a^ | 24.77±0.67^b^ | 23.21±0.66^c^ | 25.60±0.75 | 25.87±0.59 | 25.27±1.17 | 25.69±0.42 | 25.42±0.62 | 25.37±0.79 | 26.17±0.95^a^ | 26.21±0.90^a^ | 24.65±0.68^b^ |

Note: Different letters in the same line indicate significant differences among different sampling time points (*P* < 0.05).





**Figure. S1.** Melting curves of six candidate reference genes.

**Table S3.** Stability analysis of six candidate reference genes at various soybean meal levels (BestKeeper)

|  |  | Sampling time points | | | | | |
| --- | --- | --- | --- | --- | --- | --- | --- |
|  |  | 2W | | 4W | | 6W | |
| Tissues | Rank | Genes | SD | Genes | SD | Genes | SD |
| Liver | 1 | 18S rRNA | 0.33 | 18S rRNA | 0.42 | EF1a | 0.30 |
|  | 2 | β-actin | 0.38 | EF1a | 0.47 | β-actin | 0.32 |
|  | 3 | GAPDH | 0.47 | β-actin | 0.56 | B2M | 0.33 |
|  | 4 | HPRT1 | 0.55 | GAPDH | 0.58 | GAPDH | 0.34 |
|  | 5 | B2M | 0.64 | B2M | 0.60 | 18S rRNA | 0.38 |
|  | 6 | EF1a | 0.64 | HPRT1 | 0.69 | HPRT1 | 0.46 |
| Foregut | 1 | β-actin | 0.51 | 18S rRNA | 0.31 | EF1a | 0.53 |
|  | 2 | HPRT1 | 0.56 | β-actin | 0.40 | β-actin | 0.56 |
|  | 3 | EF1a | 0.66 | EF1a | 0.58 | GAPDH | 0.58 |
|  | 4 | GAPDH | 0.73 | GAPDH | 0.61 | HPRT1 | 0.65 |
|  | 5 | B2M | 0.74 | HPRT1 | 0.73 | B2M | 0.95 |
|  | 6 | 18S rRNA | 1.59 | B2M | 0.75 | 18S rRNA | 0.97 |
| Midgut | 1 | GAPDH | 0.58 | β-actin | 0.29 | 18S rRNA | 0.36 |
|  | 2 | HPRT1 | 0.59 | EF1a | 0.3 | HPRT1 | 0.57 |
|  | 3 | β-actin | 0.66 | HPRT1 | 0.38 | β-actin | 0.59 |
|  | 4 | EF1a | 0.79 | B2M | 0.42 | B2M | 0.61 |
|  | 5 | B2M | 0.87 | GAPDH | 0.49 | GAPDH | 0.74 |
|  | 6 | 18S rRNA | 1.02 | 18S rRNA | 0.57 | EF1a | 0.83 |
| Hindgut | 1 | 18S rRNA | 0.33 | 18S rRNA | 0.44 | HPRT1 | 0.59 |
|  | 2 | β-actin | 0.52 | β-actin | 0.48 | β-actin | 0.61 |
|  | 3 | HPRT1 | 0.54 | EF1a | 0.51 | 18S rRNA | 0.71 |
|  | 4 | GAPDH | 0.56 | HPRT1 | 0.53 | B2M | 0.79 |
|  | 5 | EF1a | 0.75 | GAPDH | 0.57 | EF1a | 0.92 |
|  | 6 | B2M | 0.83 | B2M | 0.88 | GAPDH | 1.02 |

**Table S4.** Stability analysis of six candidate reference genes at various soybean meal levels (NormFinder)

|  |  | Sampling time points | | | | | |
| --- | --- | --- | --- | --- | --- | --- | --- |
|  |  | 2W | | 4W | | 6W | |
| Tissues | Rank | Genes | Stability index | Genes | Stability index | Genes | Stability index |
| Liver | 1 | β-actin | 0.238 | GAPDH | 0.210 | β-actin | 0.200 |
|  | 2 | GAPDH | 0.270 | 18S rRNA | 0.318 | GAPDH | 0.295 |
|  | 3 | B2M | 0.438 | EF1a | 0.348 | B2M | 0.300 |
|  | 4 | EF1a | 0.486 | B2M | 0.374 | HPRT1 | 0.383 |
|  | 5 | 18S rRNA | 0.583 | β-actin | 0.407 | EF1a | 0.393 |
|  | 6 | HPRT1 | 0.584 | HPRT1 | 0.652 | 18S rRNA | 0.552 |
| Foregut | 1 | HPRT1 | 0.202 | β-actin | 0.344 | EF1a | 0.095 |
|  | 2 | EF1a | 0.278 | 18S rRNA | 0.419 | β-actin | 0.107 |
|  | 3 | β-actin | 0.392 | GAPDH | 0.423 | HPRT1 | 0.376 |
|  | 4 | B2M | 0.491 | EF1a | 0.442 | GAPDH | 0.504 |
|  | 5 | GAPDH | 0.603 | HPRT1 | 0.475 | B2M | 0.863 |
|  | 6 | 18S rRNA | 1.335 | B2M | 0.593 | 18S rRNA | 1.060 |
| Midgut | 1 | β-actin | 0.127 | EF1a | 0.060 | β-actin | 0.236 |
|  | 2 | HPRT1 | 0.285 | β-actin | 0.266 | B2M | 0.358 |
|  | 3 | EF1a | 0.287 | HPRT1 | 0.333 | HPRT1 | 0.457 |
|  | 4 | GAPDH | 0.549 | B2M | 0.379 | GAPDH | 0.555 |
|  | 5 | B2M | 0.572 | GAPDH | 0.470 | 18S rRNA | 0.594 |
|  | 6 | 18S rRNA | 1.209 | 18S rRNA | 0.673 | EF1a | 0.594 |
| Hindgut | 1 | β-actin | 0.160 | HPRT1 | 0.152 | β-actin | 0.170 |
|  | 2 | HPRT1 | 0.244 | EF1a | 0.180 | 18S rRNA | 0.397 |
|  | 3 | GAPDH | 0.287 | β-actin | 0.196 | EF1a | 0.479 |
|  | 4 | EF1a | 0.446 | GAPDH | 0.415 | B2M | 0.570 |
|  | 5 | B2M | 0.686 | B2M | 0.658 | HPRT1 | 0.588 |
|  | 6 | 18S rRNA | 0.805 | 18S rRNA | 0.879 | GAPDH | 0.858 |

**Table S5.** Stability analysis of six candidate reference genes at various soybean meal levels (GeNorm)

|  |  | Sampling time points | | | | | |
| --- | --- | --- | --- | --- | --- | --- | --- |
|  |  | 2W | | 4W | | 6W | |
| Tissues | Rank | Genes | M value | Genes | M value | Genes | M value |
| Liver | 1 | β-actin/GAPDH | 0.319 | 18S rRNA/EF1a | 0.402 | GAPDH/B2M | 0.298 |
|  | 2 |  |  |  |  |  |  |
|  | 3 | EF1a | 0.434 | GAPDH | 0.420 | β-actin | 0.380 |
|  | 4 | B2M | 0.490 | B2M | 0.460 | HPRT1 | 0.421 |
|  | 5 | 18S rRNA | 0.579 | β-actin | 0.473 | EF1a | 0.450 |
|  | 6 | HPRT1 | 0.626 | HPRT1 | 0.559 | 18S rRNA | 0.511 |
| Foregut | 1 | β-actin /HPRT1 | 0.461 | 18S rRNA/β-actin | 0.437 | β-actin/EF1a | 0.379 |
|  | 2 |  |  |  |  |  |  |
|  | 3 | EF1a | 0.478 | EF1a | 0.522 | GAPDH | 0.474 |
|  | 4 | B2M | 0.530 | HPRT1 | 0.564 | HPRT1 | 0.515 |
|  | 5 | GAPDH | 0.584 | GAPDH | 0.593 | B2M | 0.653 |
|  | 6 | 18S rRNA | 0.855 | B2M | 0.637 | 18S rRNA | 0.816 |
| Midgut | 1 | EF1a/HPRT1 | 0.388 | β-actin/EF1a | 0.282 | β-actin/B2M | 0.471 |
|  | 2 |  |  |  |  |  |  |
|  | 3 | β-actin | 0.405 | HPRT1 | 0.371 | HPRT1 | 0.520 |
|  | 4 | B2M | 0.497 | B2M | 0.410 | 18S rRNA | 0.550 |
|  | 5 | GAPDH | 0.555 | GAPDH | 0.459 | GAPDH | 0.633 |
|  | 6 | 18S rRNA | 0.794 | 18S rRNA | 0.555 | EF1a | 0.665 |
| Hindgut | 1 | β-actin/HPRT1 | 0.320 | β-actin/EF1a | 0.290 | 18S rRNA/β-actin | 0.426 |
|  | 2 |  |  |  |  |  |  |
|  | 3 | GAPDH | 0.392 | HPRT1 | 0.316 | HPRT1 | 0.500 |
|  | 4 | EF1a | 0.426 | GAPDH | 0.419 | EF1a | 0.559 |
|  | 5 | B2M | 0.545 | B2M | 0.501 | B2M | 0.644 |
|  | 6 | 18S rRNA | 0.659 | 18S rRNA | 0.646 | GAPDH | 0.753 |

**Table S6.** Stability analysis of six candidate reference genes at various soybean meal levels (Delta Ct)

|  |  | Sampling time points | | | | | |
| --- | --- | --- | --- | --- | --- | --- | --- |
|  |  | 2W | | 4W | | 6W | |
| Tissues | Rank | Genes | Average SD | Genes | Average SD | Genes | Average SD |
| Liver | 1 | β-actin | 0.53 | GAPDH | 0.48 | β-actin | 0.44 |
|  | 2 | GAPDH | 0.53 | 18S rRNA | 0.52 | GAPDH | 0.47 |
|  | 3 | B2M | 0.62 | EF1a | 0.53 | B2M | 0.47 |
|  | 4 | EF1a | 0.64 | B2M | 0.54 | HPRT1 | 0.52 |
|  | 5 | 18S rRNA | 0.71 | β-actin | 0.56 | EF1a | 0.53 |
|  | 6 | HPRT1 | 0.72 | HPRT1 | 0.73 | 18S rRNA | 0.63 |
| Foregut | 1 | EF1a | 0.69 | β-actin | 0.58 | β-actin | 0.64 |
|  | 2 | HPRT1 | 0.69 | 18S rRNA | 0.61 | EF1a | 0.65 |
|  | 3 | β-actin | 0.74 | GAPDH | 0.62 | HPRT1 | 0.71 |
|  | 4 | B2M | 0.78 | EF1a | 0.63 | GAPDH | 0.76 |
|  | 5 | GAPDH | 0.84 | HPRT1 | 0.65 | B2M | 0.99 |
|  | 6 | 18S rRNA | 1.40 | B2M | 0.72 | 18S rRNA | 1.14 |
| Midgut | 1 | β-actin | 0.61 | EF1a | 0.43 | β-actin | 0.57 |
|  | 2 | EF1a | 0.65 | β-actin | 0.49 | B2M | 0.61 |
|  | 3 | HPRT1 | 0.66 | HPRT1 | 0.52 | HPRT1 | 0.65 |
|  | 4 | GAPDH | 0.78 | B2M | 0.54 | GAPDH | 0.71 |
|  | 5 | B2M | 0.79 | GAPDH | 0.6 | 18S rRNA | 0.73 |
|  | 6 | 18S rRNA | 1.27 | 18S rRNA | 0.75 | EF1a | 0.73 |
| Hindgut | 1 | β-actin | 0.51 | HPRT1 | 0.51 | β-actin | 0.6 |
|  | 2 | HPRT1 | 0.56 | EF1a | 0.52 | 18S rRNA | 0.67 |
|  | 3 | GAPDH | 0.57 | β-actin | 0.53 | EF1a | 0.72 |
|  | 4 | EF1a | 0.63 | GAPDH | 0.63 | HPRT1 | 0.77 |
|  | 5 | B2M | 0.80 | B2M | 0.75 | B2M | 0.79 |
|  | 6 | 18S rRNA | 0.89 | 18S rRNA | 0.94 | GAPDH | 0.97 |

**Table S7.** Stability analysis of six candidate reference genes among different tissues (BestKeeper)

|  |  | Soybean meal levels | | | | | | | | | |
| --- | --- | --- | --- | --- | --- | --- | --- | --- | --- | --- | --- |
|  |  | 0% | | 25% | | 50% | | 75% | | 100% | |
| Sampling | Rank | Genes | SD | Genes | SD | Genes | SD | Genes | SD | Genes | SD |
| time points |  |  |  |  |  |  |  |  |  |  |  |
| 2W | 1 | HPRT1 | 0.64 | HPRT1 | 0.75 | HPRT1 | 0.57 | HPRT1 | 0.57 | HPRT1 | 0.52 |
|  | 2 | 18S rRNA | 0.84 | EF1a | 0.84 | EF1a | 0.62 | EF1a | 0.65 | EF1a | 0.82 |
|  | 3 | EF1a | 0.86 | GAPDH | 0.99 | GAPDH | 0.84 | 18S rRNA | 0.83 | β-actin | 1.01 |
|  | 4 | β-actin | 1.13 | β-actin | 1.19 | 18S rRNA | 1.04 | β-actin | 0.92 | 18S rRNA | 1.04 |
|  | 5 | GAPDH | 1.15 | 18S rRNA | 1.35 | β-actin | 1.05 | GAPDH | 0.95 | GAPDH | 1.20 |
|  | 6 | B2M | 1.81 | B2M | 1.86 | B2M | 1.52 | B2M | 1.42 | B2M | 1.64 |
| 4W | 1 | 18S rRNA | 0.56 | 18S rRNA | 0.32 | 18S rRNA | 0.42 | 18S rRNA | 0.48 | 18S rRNA | 0.60 |
|  | 2 | HPRT1 | 0.63 | HPRT1 | 0.56 | EF1a | 0.71 | HPRT1 | 0.53 | EF1a | 0.61 |
|  | 3 | EF1a | 0.76 | GAPDH | 0.59 | HPRT1 | 0.73 | EF1a | 0.64 | HPRT1 | 0.67 |
|  | 4 | GAPDH | 0.83 | β-actin | 0.80 | β-actin | 0.88 | GAPDH | 0.73 | β-actin | 0.89 |
|  | 5 | β-actin | 0.94 | EF1a | 0.80 | GAPDH | 0.95 | β-actin | 1.01 | GAPDH | 0.97 |
|  | 6 | B2M | 1.50 | B2M | 1.44 | B2M | 1.47 | B2M | 1.42 | B2M | 1.35 |
| 6W | 1 | GAPDH | 0.60 | EF1a | 0.55 | EF1a | 0.75 | 18S rRNA | 0.71 | EF1a | 0.71 |
|  | 2 | EF1a | 0.63 | 18S rRNA | 0.74 | GAPDH | 0.86 | EF1a | 0.79 | 18S rRNA | 0.82 |
|  | 3 | 18S rRNA | 0.66 | GAPDH | 0.86 | 18S rRNA | 0.86 | HPRT1 | 0.96 | HPRT1 | 0.94 |
|  | 4 | β-actin | 0.89 | β-actin | 0.90 | HPRT1 | 0.87 | β-actin | 0.97 | GAPDH | 0.98 |
|  | 5 | HPRT1 | 0.92 | HPRT1 | 0.99 | β-actin | 1.09 | GAPDH | 1.34 | β-actin | 1.03 |
|  | 6 | B2M | 1.74 | B2M | 1.38 | B2M | 1.72 | B2M | 1.47 | B2M | 1.61 |

**Table S8.** Stability analysis of six candidate reference genes among different tissues (NormFinder)

|  |  | Soybean meal levels | | | | | | | | | |
| --- | --- | --- | --- | --- | --- | --- | --- | --- | --- | --- | --- |
|  |  | 0% | | 25% | | 50% | | 75% | | 100% | |
| Sampling | Rank | Genes | Stability index  index | Genes | Stability index | Genes | Stability index | Genes | Stability index | Genes | Stability index |
| time points |  |  |  |  |  |  |  |  |  |  |  |
| 2W | 1 | EF1a | 0.078 | HPRT1 | 0.099 | EF1a | 0.359 | HPRT1 | 0.304 | HPRT1 | 0.141 |
|  | 2 | HPRT1 | 0.099 | EF1a | 0.177 | HPRT1 | 0.491 | EF1a | 0.384 | EF1a | 0.317 |
|  | 3 | β-actin | 0.629 | β-actin | 0.691 | β-actin | 0.736 | β-actin | 0.699 | β-actin | 0.840 |
|  | 4 | 18S rRNA | 0.921 | 18S rRNA | 0.889 | 18S rRNA | 1.418 | 18S rRNA | 1.027 | 18S rRNA | 1.298 |
|  | 5 | B2M | 1.690 | GAPDH | 1.600 | B2M | 1.526 | B2M | 1.380 | B2M | 1.402 |
|  | 6 | GAPDH | 1.782 | B2M | 1.606 | GAPDH | 1.645 | GAPDH | 1.559 | GAPDH | 1.562 |
| 4W | 1 | EF1a | 0.258 | EF1a | 0.345 | EF1a | 0.226 | EF1a | 0.298 | EF1a | 0.334 |
|  | 2 | β-actin | 0.619 | β-actin | 0.363 | HPRT1 | 0.518 | 18S rRNA | 0.568 | 18S rRNA | 0.557 |
|  | 3 | 18S rRNA | 0.866 | HPRT1 | 0.475 | β-actin | 0.612 | β-actin | 0.871 | HPRT1 | 0.886 |
|  | 4 | HPRT1 | 1.013 | 18S rRNA | 0.625 | 18S rRNA | 0.817 | HPRT1 | 1.006 | β-actin | 0.901 |
|  | 5 | GAPDH | 1.279 | GAPDH | 1.262 | B2M | 1.327 | GAPDH | 1.292 | B2M | 1.319 |
|  | 6 | B2M | 1.507 | B2M | 1.334 | GAPDH | 1.497 | B2M | 1.376 | GAPDH | 1.394 |
| 6W | 1 | EF1a | 0.265 | EF1a | 0.387 | EF1a | 0.497 | EF1a | 0.157 | EF1a | 0.413 |
|  | 2 | β-actin | 0.513 | 18S rRNA | 0.725 | β-actin | 0.831 | 18S rRNA | 0.813 | β-actin | 0.795 |
|  | 3 | GAPDH | 0.579 | β-actin | 0.810 | 18S rRNA | 1.039 | β-actin | 0.830 | 18S rRNA | 0.984 |
|  | 4 | 18S rRNA | 0.780 | GAPDH | 1.102 | GAPDH | 1.282 | HPRT1 | 1.287 | GAPDH | 1.153 |
|  | 5 | HPRT1 | 1.639 | HPRT1 | 1.578 | HPRT1 | 1.333 | B2M | 1.601 | HPRT1 | 1.397 |
|  | 6 | B2M | 1.904 | B2M | 1.722 | B2M | 1.831 | GAPDH | 1.626 | B2M | 1.529 |

**Table S9.** Stability analysis of six candidate reference genes among different tissues (GeNorm)

|  |  | Soybean meal levels | | | | | | | | | | |
| --- | --- | --- | --- | --- | --- | --- | --- | --- | --- | --- | --- | --- |
| Sampling |  | 0% | | 25% | | 50% | | 75% | | 100% | | |
|  | Rank | Genes | M value | Genes | M value | Genes | M value | Genes | M value | Genes | M  value |  |
| time points |  |  |  |  |  |  |  |  |  |  |  |  |
| 2W | 1 | EF1a \| HPRT1 | 0.689 | EF1a \| HPRT1 | 0.354 | EF1a \| HPRT1 | 0.622 | EF1a \| HPRT1 | 0.718 | EF1a \| HPRT1 | 0.461 |  |
|  | 2 |  |  |  |  |  |  |  |  |  |  |  |
|  | 3 | β-actin | 0.860 | β-actin | 0.712 | β-actin | 0.850 | β-actin | 0.803 | β-actin | 0.774 |  |
|  | 4 | 18S rRNA | 0.970 | 18S rRNA | 0.933 | B2M | 1.087 | B2M | 0.999 | B2M | 1.000 |  |
|  | 5 | B2M | 1.221 | B2M | 1.156 | 18S rRNA | 1.374 | 18S rRNA | 1.173 | 18S rRNA | 1.264 |  |
|  | 6 | GAPDH | 1.458 | GAPDH | 1.350 | GAPDH | 1.544 | GAPDH | 1.358 | GAPDH | 1.435 |  |
| 4W | 1 | β-actin \| EF1a | 0.603 | β-actin \| EF1a | 0.407 | β-actin \| EF1a | 0.452 | GAPDH \| HPRT1 | 0.528 | β-actin \| EF1a | 0.667 |  |
|  | 2 |  |  |  |  |  |  |  |  |  |  |  |
|  | 3 | 18S rRNA | 0.877 | HPRT1 | 0.696 | HPRT1 | 0.807 | 18S rRNA | 0.909 | 18S rRNA | 0.850 |  |
|  | 4 | HPRT1 | 1.098 | 18S rRNA | 0.771 | 18S rRNA | 0.912 | EF1a | 1.018 | B2M | 0.994 |  |
|  | 5 | GAPDH | 1.188 | GAPDH | 0.945 | B2M | 1.053 | β-actin | 1.142 | HPRT1 | 1.137 |  |
|  | 6 | B2M | 1.348 | B2M | 1.111 | GAPDH | 1.243 | B2M | 1.267 | GAPDH | 1.278 |  |
| 6W | 1 | GAPDH \| EF1a | 0.531 | 18S rRNA \| EF1a | 0.774 | GAPDH \| HPRT1 | 1.046 | β-actin \| B2M | 0.810 | β-actin \| EF1a | 0.827 |  |
|  | 2 |  |  |  |  |  |  |  |  |  |  |  |
|  | 3 | β-actin | 0.835 | β-actin | 0.922 | EF1a | 1.130 | EF1a | 1.116 | 18S rRNA | 1.037 |  |
|  | 4 | 18S rRNA | 0.951 | GAPDH | 1.145 | 18S rRNA | 1.267 | 18S rRNA | 1.258 | GAPDH | 1.246 |  |
|  | 5 | HPRT1 | 1.183 | HPRT1 | 1.315 | β-actin | 1.388 | HPRT1 | 1.427 | HPRT1 | 1.352 |  |
|  | 6 | B2M | 1.459 | B2M | 1.504 | B2M | 1.588 | GAPDH | 1.585 | B2M | 1.482 |  |

**Table S10.** Stability analysis of six candidate reference genes among different tissues (Delta Ct)

|  |  | Soybean meal levels | | | | | | | | | | |
| --- | --- | --- | --- | --- | --- | --- | --- | --- | --- | --- | --- | --- |
|  |  | 0% | | 25% | | 50% | | 75% | | 100% | | |
| Sampling | Rank | Genes | Average SD | Genes | Average SD | Genes | Average SD | Genes | Average SD | Genes | Average SD |  |
| time points |  |  |  |  |  |  |  |  |  |  |  |  |
| 2W | 1 | HPRT1 | 1.15 | EF1a | 1.03 | EF1a | 1.24 | HPRT1 | 1.10 | HPRT1 | 1.12 |  |
|  | 2 | EF1a | 1.16 | HPRT1 | 1.05 | HPRT1 | 1.27 | EF1a | 1.12 | EF1a | 1.14 |  |
|  | 3 | β-actin | 1.26 | β-actin | 1.20 | β-actin | 1.33 | β-actin | 1.20 | β-actin | 1.30 |  |
|  | 4 | 18S rRNA | 1.40 | 18S rRNA | 1.35 | B2M | 1.76 | 18S rRNA | 1.42 | B2M | 1.63 |  |
|  | 5 | B2M | 1.85 | B2M | 1.73 | 18S rRNA | 1.78 | B2M | 1.57 | 18S rRNA | 1.64 |  |
|  | 6 | GAPDH | 1.93 | GAPDH | 1.74 | GAPDH | 1.88 | GAPDH | 1.73 | GAPDH | 1.78 |  |
| 4W | 1 | EF1a | 1.09 | EF1a | 0.91 | EF1a | 0.96 | EF1a | 0.99 | EF1a | 0.98 |  |
|  | 2 | β-actin | 1.17 | β-actin | 0.91 | β-actin | 1.07 | 18S rRNA | 1.14 | 18S rRNA | 1.13 |  |
|  | 3 | 18S rRNA | 1.31 | HPRT1 | 0.97 | HPRT1 | 1.10 | β-actin | 1.21 | β-actin | 1.24 |  |
|  | 4 | HPRT1 | 1.34 | 18S rRNA | 1.04 | 18S rRNA | 1.23 | HPRT1 | 1.28 | HPRT1 | 1.25 |  |
|  | 5 | GAPDH | 1.50 | GAPDH | 1.39 | B2M | 1.48 | GAPDH | 1.45 | B2M | 1.50 |  |
|  | 6 | B2M | 1.67 | B2M | 1.44 | GAPDH | 1.63 | B2M | 1.52 | GAPDH | 1.56 |  |
| 6W | 1 | EF1a | 1.12 | EF1a | 1.14 | EF1a | 1.24 | EF1a | 1.28 | EF1a | 1.15 |  |
|  | 2 | GAPDH | 1.23 | 18S rRNA | 1.35 | β-actin | 1.43 | β-actin | 1.40 | β-actin | 1.33 |  |
|  | 3 | β-actin | 1.25 | β-actin | 1.35 | 18S rRNA | 1.56 | 18S rRNA | 1.44 | 18S rRNA | 1.45 |  |
|  | 4 | 18S rRNA | 1.34 | GAPDH | 1.51 | GAPDH | 1.64 | HPRT1 | 1.67 | GAPDH | 1.54 |  |
|  | 5 | HPRT1 | 1.81 | HPRT1 | 1.79 | HPRT1 | 1.67 | B2M | 1.82 | HPRT1 | 1.68 |  |
|  | 6 | B2M | 2.01 | B2M | 1.88 | B2M | 1.99 | GAPDH | 1.90 | B2M | 1.74 |  |

**Table S11.** Stability analysis of six candidate reference genes in different sampling time points (BestKeeper)

|  |  | Soybean meal levels | | | | | | | | | |
| --- | --- | --- | --- | --- | --- | --- | --- | --- | --- | --- | --- |
|  |  | 0% | | 25% | | 50% | | 75% | | 100% | |
| Tissues | Rank | Genes | SD | Genes | SD | Genes | SD | Genes | SD | Genes | SD |
| Liver | 1 | β-actin | 0.46 | B2M | 0.52 | β-actin | 0.43 | B2M | 0.41 | B2M | 0.43 |
|  | 2 | B2M | 0.48 | β-actin | 0.55 | B2M | 0.53 | β-actin | 0.46 | EF1a | 0.48 |
|  | 3 | 18S rRNA | 0.63 | 18S rRNA | 0.68 | 18S rRNA | 0.62 | GAPDH | 0.50 | β-actin | 0.51 |
|  | 4 | EF1a | 0.68 | GAPDH | 0.79 | GAPDH | 0.64 | EF1a | 0.72 | GAPDH | 0.60 |
|  | 5 | GAPDH | 0.77 | EF1a | 1.01 | EF1a | 0.70 | 18S rRNA | 0.76 | 18S rRNA | 0.63 |
|  | 6 | HPRT1 | 1.33 | HPRT1 | 1.59 | HPRT1 | 1.31 | HPRT1 | 1.22 | HPRT1 | 1.10 |
| Foregut | 1 | β-actin | 0.54 | β-actin | 0.50 | β-actin | 0.47 | HPRT1 | 0.56 | β-actin | 0.49 |
|  | 2 | HPRT1 | 0.60 | GAPDH | 0.57 | EF1a | 0.53 | β-actin | 0.61 | EF1a | 0.50 |
|  | 3 | EF1a | 0.62 | EF1a | 0.64 | GAPDH | 0.61 | EF1a | 0.66 | GAPDH | 0.68 |
|  | 4 | GAPDH | 0.64 | HPRT1 | 0.65 | HPRT1 | 0.65 | GAPDH | 0.79 | HPRT1 | 0.70 |
|  | 5 | B2M | 0.66 | B2M | 1.08 | B2M | 0.76 | B2M | 0.81 | B2M | 1.05 |
|  | 6 | 18S rRNA | 1.08 | 18S rRNA | 1.62 | 18S rRNA | 1.61 | 18S rRNA | 1.58 | 18S rRNA | 1.82 |
| Midgut | 1 | HPRT1 | 0.63 | GAPDH | 0.53 | 18S rRNA | 0.42 | β-actin | 0.37 | 18S rRNA | 0.42 |
|  | 2 | β-actin | 0.79 | HPRT1 | 0.59 | B2M | 0.54 | HPRT1 | 0.41 | HPRT1 | 0.46 |
|  | 3 | B2M | 0.83 | β-actin | 0.77 | GAPDH | 0.56 | B2M | 0.45 | β-actin | 0.55 |
|  | 4 | GAPDH | 0.92 | EF1a | 0.81 | HPRT1 | 0.56 | EF1a | 0.50 | B2M | 0.58 |
|  | 5 | EF1a | 0.97 | 18S rRNA | 0.86 | β-actin | 0.66 | GAPDH | 0.50 | GAPDH | 0.65 |
|  | 6 | 18S rRNA | 1.11 | B2M | 0.87 | EF1a | 0.88 | 18S rRNA | 0.53 | EF1a | 0.88 |
| Hindgut | 1 | β-actin | 0.43 | GAPDH | 0.37 | B2M | 0.58 | β-actin | 0.54 | 18S rRNA | 0.66 |
|  | 2 | GAPDH | 0.51 | HPRT1 | 0.45 | β-actin | 0.60 | 18S rRNA | 0.67 | β-actin | 0.72 |
|  | 3 | HPRT1 | 0.59 | β-actin | 0.51 | GAPDH | 0.62 | HPRT1 | 0.70 | HPRT1 | 0.87 |
|  | 4 | 18S rRNA | 0.63 | B2M | 0.58 | EF1a | 0.64 | B2M | 0.78 | EF1a | 0.88 |
|  | 5 | EF1a | 0.69 | EF1a | 0.59 | HPRT1 | 0.68 | EF1a | 0.84 | GAPDH | 0.99 |
|  | 6 | B2M | 0.72 | 18S rRNA | 0.67 | 18S rRNA | 0.77 | GAPDH | 1.15 | B2M | 1.22 |

**Table S12.** Stability analysis of six candidate reference genes in different sampling time points (NormFinder)

|  |  | Soybean meal levels | | | | | | | | | |
| --- | --- | --- | --- | --- | --- | --- | --- | --- | --- | --- | --- |
|  |  | 0% | | 25% | | 50% | | 75% | | 100% | |
| Tissues | Rank | Genes | Stability index | Genes | Stability index | Genes | Stability index | Genes | Stability index | Genes | Stability index |
| Liver | 1 | β-actin | 0.232 | β-actin | 0.278 | β-actin | 0.198 | β-actin | 0.263 | β-actin | 0.187 |
|  | 2 | B2M | 0.518 | 18S rRNA | 0.446 | 18S rRNA | 0.493 | B2M | 0.485 | B2M | 0.354 |
|  | 3 | 18S rRNA | 0.536 | B2M | 0.547 | B2M | 0.563 | EF1a | 0.519 | EF1a | 0.411 |
|  | 4 | EF1a | 0.733 | EF1a | 0.575 | EF1a | 0.572 | 18S rRNA | 0.669 | 18S rRNA | 0.609 |
|  | 5 | GAPDH | 0.918 | GAPDH | 0.593 | GAPDH | 0.728 | GAPDH | 0.769 | GAPDH | 0.683 |
|  | 6 | HPRT1 | 1.544 | HPRT1 | 1.463 | HPRT1 | 1.472 | HPRT1 | 1.662 | HPRT1 | 1.474 |
| Foregut | 1 | β-actin | 0.297 | β-actin | 0.231 | β-actin | 0.193 | EF1a | 0.191 | EF1a | 0.114 |
|  | 2 | EF1a | 0.381 | EF1a | 0.256 | EF1a | 0.193 | β-actin | 0.279 | β-actin | 0.307 |
|  | 3 | HPRT1 | 0.442 | GAPDH | 0.369 | GAPDH | 0.514 | B2M | 0.452 | GAPDH | 0.412 |
|  | 4 | GAPDH | 0.455 | HPRT1 | 0.856 | B2M | 0.610 | HPRT1 | 0.557 | HPRT1 | 0.540 |
|  | 5 | B2M | 0.772 | B2M | 0.885 | HPRT1 | 0.826 | GAPDH | 0.568 | B2M | 0.787 |
|  | 6 | 18S rRNA | 1.286 | 18S rRNA | 1.437 | 18S rRNA | 1.826 | 18S rRNA | 1.531 | 18S rRNA | 1.840 |
| Midgut | 1 | β-actin | 0.233 | β-actin | 0.129 | B2M | 0.178 | β-actin | 0.319 | β-actin | 0.162 |
|  | 2 | HPRT1 | 0.383 | EF1a | 0.259 | β-actin | 0.345 | EF1a | 0.395 | HPRT1 | 0.360 |
|  | 3 | EF1a | 0.448 | HPRT1 | 0.403 | GAPDH | 0.381 | B2M | 0.414 | B2M | 0.492 |
|  | 4 | GAPDH | 0.606 | B2M | 0.518 | HPRT1 | 0.532 | HPRT1 | 0.454 | EF1a | 0.495 |
|  | 5 | B2M | 0.664 | GAPDH | 0.566 | EF1a | 0.600 | GAPDH | 0.520 | GAPDH | 0.497 |
|  | 6 | 18S rRNA | 1.236 | 18S rRNA | 0.856 | 18S rRNA | 0.760 | 18S rRNA | 0.679 | 18S rRNA | 0.576 |
| Hindgut | 1 | GAPDH | 0.181 | β-actin | 0.098 | β-actin | 0.191 | β-actin | 0.303 | EF1a | 0.314 |
|  | 2 | HPRT1 | 0.200 | HPRT1 | 0.177 | EF1a | 0.368 | B2M | 0.333 | β-actin | 0.328 |
|  | 3 | β-actin | 0.249 | EF1a | 0.336 | HPRT1 | 0.469 | EF1a | 0.602 | HPRT1 | 0.614 |
|  | 4 | EF1a | 0.546 | B2M | 0.467 | B2M | 0.555 | HPRT1 | 0.832 | GAPDH | 0.656 |
|  | 5 | B2M | 0.587 | GAPDH | 0.624 | GAPDH | 0.600 | 18S rRNA | 0.926 | B2M | 0.730 |
|  | 6 | 18S rRNA | 0.873 | 18S rRNA | 0.808 | 18S rRNA | 0.954 | GAPDH | 1.186 | 18S rRNA | 0.799 |

**Table S13.** Stability analysis of six candidate reference genes in different sampling time points (GeNorm)

|  |  | Soybean meal levels | | | | | | | | | |
| --- | --- | --- | --- | --- | --- | --- | --- | --- | --- | --- | --- |
|  |  | 0% | | 25% | | 50% | | 75% | | 100% | |
| Tissues | Rank | Genes | M value | Genes | M value | Genes | M value | Genes | M value | Genes | M  value |
| Liver | 1 | β-actin \| B2M | 0.464 | β-actin \| B2M | 0.463 | β-actin \| B2M | 0.538 | β-actin \| GAPDH | 0.509 | β-actin \| B2M | 0.375 |
|  | 2 |  |  |  |  |  |  |  |  |  |  |
|  | 3 | GAPDH | 0.675 | GAPDH | 0.515 | GAPDH | 0.607 | B2M | 0.611 | GAPDH | 0.511 |
|  | 4 | EF1a | 0.790 | 18S rRNA | 0.646 | 18S rRNA | 0.711 | EF1a | 0.669 | EF1a | 0.572 |
|  | 5 | 18S rRNA | 0.836 | EF1a | 0.691 | EF1a | 0.748 | 18S rRNA | 0.778 | 18S rRNA | 0.643 |
|  | 6 | HPRT1 | 1.103 | HPRT1 | 0.975 | HPRT1 | 1.018 | HPRT1 | 1.102 | HPRT1 | 0.941 |
| Foregut | 1 | β-actin \| HPRT1 | 0.566 | β-actin \| GAPDH | 0.462 | β-actin \| EF1a | 0.385 | β-actin \| EF1a | 0.428 | β-actin \| EF1a | 0.373 |
|  | 2 |  |  |  |  |  |  |  |  |  |  |
|  | 3 | GAPDH | 0.615 | EF1a | 0.543 | GAPDH | 0.521 | B2M | 0.468 | HPRT1 | 0.475 |
|  | 4 | EF1a | 0.645 | HPRT1 | 0.667 | HPRT1 | 0.610 | HPRT1 | 0.537 | GAPDH | 0.595 |
|  | 5 | B2M | 0.696 | B2M | 0.800 | B2M | 0.680 | GAPDH | 0.605 | B2M | 0.673 |
|  | 6 | 18S rRNA | 0.919 | 18S rRNA | 1.045 | 18S rRNA | 1.081 | 18S rRNA | 0.933 | 18S rRNA | 1.081 |
| Midgut | 1 | β-actin \| HPRT1 | 0.466 | β-actin \| EF1a | 0.292 | GAPDH \| B2M | 0.444 | β-actin \| EF1a | 0.413 | β-actin \| EF1a | 0.428 |
|  | 2 |  |  |  |  |  |  |  |  |  |  |
|  | 3 | GAPDH | 0.551 | HPRT1 | 0.484 | β-actin | 0.485 | B2M | 0.534 | GAPDH | 0.489 |
|  | 4 | B2M | 0.599 | GAPDH | 0.539 | EF1a | 0.540 | HPRT1 | 0.556 | HPRT1 | 0.544 |
|  | 5 | EF1a | 0.660 | B2M | 0.567 | HPRT1 | 0.601 | GAPDH | 0.588 | B2M | 0.585 |
|  | 6 | 18S rRNA | 0.876 | 18S rRNA | 0.689 | 18S rRNA | 0.689 | 18S rRNA | 0.659 | 18S rRNA | 0.625 |
| Hindgut | 1 | β-actin \| GAPDH | 0.362 | β-actin \| EF1a | 0.291 | β-actin \| EF1a | 0.381 | β-actin \| B2M | 0.616 | β-actin \| EF1a | 0.416 |
|  | 2 |  |  |  |  |  |  |  |  |  |  |
|  | 3 | HPRT1 | 0.399 | HPRT1 | 0.362 | GAPDH | 0.436 | HPRT1 | 0.775 | GAPDH | 0.603 |
|  | 4 | EF1a | 0.470 | B2M | 0.407 | B2M | 0.540 | 18S rRNA | 0.819 | B2M | 0.685 |
|  | 5 | B2M | 0.530 | GAPDH | 0.527 | HPRT1 | 0.619 | EF1a | 0.872 | HPRT1 | 0.765 |
|  | 6 | 18S rRNA | 0.666 | 18S rRNA | 0.647 | 18S rRNA | 0.759 | GAPDH | 1.013 | 18S rRNA | 0.828 |

**Table S14.** Stability analysis of six candidate reference genes in different sampling time points (Delta Ct)

|  |  | Soybean meal levels | | | | | | | | | | |
| --- | --- | --- | --- | --- | --- | --- | --- | --- | --- | --- | --- | --- |
|  |  | 0% | | 25% | | 50% | | 75% | | 100% | | |
| Tissues | Rank | Genes | Average SD | Genes | Average SD | Genes | Average SD | Genes | Average SD | Genes | Average SD |  |
| Liver | 1 | β-actin | 0.82 | β-actin | 0.79 | β-actin | 0.79 | β-actin | 0.85 | β-actin | 0.70 |  |
|  | 2 | B2M | 0.97 | 18S rRNA | 0.86 | 18S rRNA | 0.92 | EF1a | 0.96 | B2M | 0.80 |  |
|  | 3 | 18S rRNA | 1.00 | B2M | 0.87 | B2M | 0.93 | B2M | 0.96 | EF1a | 0.80 |  |
|  | 4 | EF1a | 1.05 | GAPDH | 0.88 | EF1a | 0.95 | GAPDH | 1.02 | GAPDH | 0.90 |  |
|  | 5 | GAPDH | 1.13 | EF1a | 0.91 | GAPDH | 0.96 | 18S rRNA | 1.07 | 18S rRNA | 0.91 |  |
|  | 6 | HPRT1 | 1.64 | HPRT1 | 1.54 | HPRT1 | 1.56 | HPRT1 | 1.75 | HPRT1 | 1.54 |  |
| Foregut | 1 | β-actin | 0.76 | β-actin | 0.80 | β-actin | 0.80 | EF1a | 0.72 | EF1a | 0.83 |  |
|  | 2 | GAPDH | 0.80 | EF1a | 0.86 | EF1a | 0.81 | β-actin | 0.75 | β-actin | 0.86 |  |
|  | 3 | EF1a | 0.81 | GAPDH | 0.86 | GAPDH | 0.94 | B2M | 0.81 | HPRT1 | 0.90 |  |
|  | 4 | HPRT1 | 0.82 | HPRT1 | 1.08 | B2M | 1.00 | HPRT1 | 0.85 | GAPDH | 0.94 |  |
|  | 5 | B2M | 0.96 | B2M | 1.14 | HPRT1 | 1.06 | GAPDH | 0.89 | B2M | 1.06 |  |
|  | 6 | 18S rRNA | 1.37 | 18S rRNA | 1.54 | 18S rRNA | 1.88 | 18S rRNA | 1.59 | 18S rRNA | 1.90 |  |
| Midgut | 1 | β-actin | 0.66 | β-actin | 0.54 | B2M | 0.56 | β-actin | 0.59 | β-actin | 0.51 |  |
|  | 2 | HPRT1 | 0.77 | EF1a | 0.59 | β-actin | 0.61 | EF1a | 0.61 | HPRT1 | 0.58 |  |
|  | 3 | EF1a | 0.81 | HPRT1 | 0.65 | GAPDH | 0.63 | B2M | 0.62 | EF1a | 0.65 |  |
|  | 4 | GAPDH | 0.83 | B2M | 0.70 | HPRT1 | 0.72 | HPRT1 | 0.65 | GAPDH | 0.66 |  |
|  | 5 | B2M | 0.87 | GAPDH | 0.72 | EF1a | 0.74 | GAPDH | 0.68 | B2M | 0.66 |  |
|  | 6 | 18S rRNA | 1.31 | 18S rRNA | 0.93 | 18S rRNA | 0.86 | 18S rRNA | 0.80 | 18S rRNA | 0.71 |  |
| Hindgut | 1 | GAPDH | 0.51 | β-actin | 0.50 | β-actin | 0.59 | β-actin | 0.79 | EF1a | 0.69 |  |
|  | 2 | HPRT1 | 0.56 | HPRT1 | 0.54 | EF1a | 0.66 | B2M | 0.86 | β-actin | 0.71 |  |
|  | 3 | β-actin | 0.56 | EF1a | 0.57 | HPRT1 | 0.74 | EF1a | 0.96 | HPRT1 | 0.84 |  |
|  | 4 | EF1a | 0.69 | B2M | 0.63 | GAPDH | 0.76 | HPRT1 | 1.06 | GAPDH | 0.87 |  |
|  | 5 | B2M | 0.73 | GAPDH | 0.76 | B2M | 0.76 | 18S rRNA | 1.11 | B2M | 0.91 |  |
|  | 6 | 18S rRNA | 0.94 | 18S rRNA | 0.89 | 18S rRNA | 1.04 | GAPDH | 1.30 | 18S rRNA | 0.95 |  |
